# Modulation of Gut Microbiota Composition and Microbial Phenolic Catabolism of Phenolic Compounds from *Achillea millefolium* L. and *Origanum majorana* L.

**DOI:** 10.1021/acs.jafc.4c07910

**Published:** 2024-12-19

**Authors:** Irene Fernandez-Jalao, María de las Nieves Siles-Sánchez, Susana Santoyo, Alba Tamargo, Edgard Relaño de la Guía, Natalia Molinero, Victoria Moreno-Arribas, Laura Jaime

**Affiliations:** † Departmental Section of Food Science, Faculty of Science, 16722 Universidad Autónoma de Madrid, Madrid 28049, Spain; ‡ Department of Production and Characterization of Novel Food, Food Science Research Institute (CIAL), CEI UAM+CSIC, Madrid 28049, Spain; § Department of Food Biotechnology and Microbiology, 370420 Food Science Research Institute (CIAL), CEI UAM+CSIC, Madrid 28049, Spain

**Keywords:** *Achillea millefolium* L, *in vitro* colonic fermentation, microbial phenolic catabolism, *Origanum majorana* L, phenolic compounds, prebiotic-like effect

## Abstract

The impact of the nonbioaccessible fraction of two phenolic-rich extracts from *Achillea millefolium* L. (yarrow) and *Origanum majorana* L. (marjoram) on the modulation of the human gut microbiota was investigated *in vitro*. Microbial metabolism of the phenolic compounds was also addressed. In general, phenolic acids or *O*-glycosidic flavones quickly disappeared, in contrast to methoxy- or *C*-glycosidic flavonoids. This colonic metabolism yielded phloroglucinol, 3,4-dimethoxyphenylacetic acid, 3-(4-hydroxyphenyl)-propionic acid, and 4-hydroxybenzoic acid as the main metabolites of the microbial catabolism of rosmarinic acid or caffeoylquinic acids, among others. The 16S rRNA gene sequencing showed that the most promising modulatory effect was related to the increase in *Bifidobacterium* spp., *Collinsella* spp., *Romboutsia*, and *Akkermansia muciniphila* for both plant extracts, along with *Blautia* spp. and *Dialister* for yarrow extract. This beneficial modulation was accompanied by the increase in butyric acid production, highlighting the potential prebiotic-like effect on the gut microbiota of these two previously unstudied edible plants.

## Graphical abstract

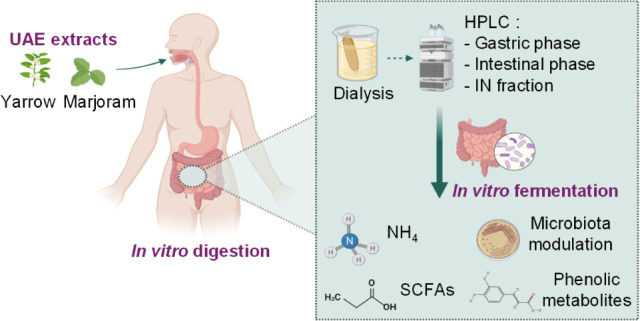

## 1 Introduction

The human gut microbiota comprises a complex ecosystem of more than 160 species of microorganisms that plays a key role in the host’s health.^1^ The well-documented association between a balanced microbiota and its protective health effects makes it a hot topic for researchers.^2,3^ In this context, the scientific community joins efforts to look for food sources that can be used as prebiotic-like ingredients.^4^ Regarding compounds, other than nondigestible carbohydrates, which can exert this modulatory function, (poly)­phenols stand out as promising ingredients.^5,6^

However, it should be considered that phenolic compounds (PCs) may undergo strong modifications during gastrointestinal digestion (GID), mainly attributed to pH conditions and enzymatic activity, leading to their degradation or transformation in other products.^7,8^ In general, PCs have low bioaccessibility and bioavailability rates, so that most of them reach the colon where they might be metabolized. The impact of dietary poly­(phenols) on human health should consider the two-way interaction between poly­(phenols) and gut microbiota: gut microbiota degrades poly­(phenols) into absorbable metabolites, and in turn, poly­(phenols) and/or their generated metabolites seem to modulate the composition and metabolic functionality of gut microbiota.^9,10^

*Achillea millefolium* L. (yarrow) and *Origanum majorana* L. (marjoram) are edible plants consumed as part of the diet commonly as culinary herbs or herbal teas but also as dietary supplements and nutraceuticals. Their composition is characterized by the presence of phenolic compounds, fats, and essential oil components, among others.^11,12^ On the other hand, some of the PCs present in these plants (caffeic acid, caffeoylquinic acids (CQAs), rosmarinic acid, or apigenin) have shown modulatory effects on gut microbiota through the increase in beneficial bacteria (*Bifidobacterium*, *Akkermansia muciniphila*, and *Faecalibacterium prausnitzii*) or the inhibition of pathogen growth (*Enterococcus caccae* or *Clostridium perfringens*).^13–15^ However, to the best of our knowledge, there are no previous studies focused on the gut modulation effect of the phenolic compounds obtained from both plants or the microbial catabolism of most of the phenolic compounds of these edible plants.

Therefore, the aim of this work was to evaluate the potential gut-microbiome-modulating properties of the phenolic compounds of two phenolic-rich extracts from yarrow and marjoram. For this purpose, the bioaccessibility of PCs was calculated after an *in vitro* GID. Following a microbiome approach, changes in microbial populations were evaluated by both the microbial plate count and 16S rRNA gene sequence analysis. Moreover, microbial metabolic activity was determined based on the disappearance of the parent PCs and the production of phenolic metabolites, ammonium, and short-chain fatty acids (SCFAs).

## 2 Materials and Methods

### 2.1 Reagents

α-Amylase (A3306), lipase from *Rhizopus oryzae* (80612), pepsin from gastric porcine mucosa (P7012), porcine bile salts (B8631), pancreatin (P7545, 8xUSP), dimethyl sulfoxide (DMSO), and ammonium standard were obtained from Sigma-Aldrich (St. Louis, MO, USA). Ethanol (99.5%), potassium phosphate monobasic, sodium chloride, magnesium chloride hexahydrate, and calcium chloride were purchased from Panreac (Barcelona, Spain). Ammonium carbonate and potassium chloride were purchased from Merck (Darmstadt, Germany). Formic acid (99%) was acquired from Acros Organics (Madrid, Spain), and acetonitrile (HPLC grade) was acquired from Macron Fine Chemicals (Madrid, Spain).

### 2.2 Sample Preparation and Ultrasound-Assisted Extraction

Dried *Achillea millefolium* L. aerial parts from Bulgaria and leaves of *Origanum majorana* L. from Egypt were purchased from a specialized herbalist’s shop (Plantafarm S.A, León, Spain, and Murciana de Herboristera, Murcia, Spain, respectively). The samples were ground (Grindomix GM 200, Retsch, Spain) and sieved to obtain a homogeneous particle size (500–250 μm).

Sample extraction was conducted with pure ethanol using a Branson 450 ultrasonic device (Branson Ultrasonic, Danbury, CT, USA) according to the methodology previously described.^16^ Briefly, ground millefolium and marjoram (40 g) were mixed with pure ethanol (1:10, w:v) and submitted to extraction for 20 min at 50 °C with continuous stirring and 60% amplitude using a probe of 1/2’ diameter. After that, the samples were filtered under vacuum, and ethanol was removed using a rotating evaporator (IKA RV 10, Madrid, Spain). Dried extracts were maintained at 4 °C in the darkness until use.

### 2.3 *In Vitro* Gastrointestinal Digestion

GID was performed according to the INFOGEST standardized protocol,^17^ replacing rabbit gastric extract with lipase from *Rhizopus oryzae*.^18^ Prior to digestion, 200 mg of dried extract was dispersed in 5 mL of ultrapure water with gentle manual agitation. Following the mentioned GID protocol, the oral phase was completed to 10 mL by adding 4 mL of salivary stock solution (SSF), 25 μL of CaCl_2_ 0.3M, 750 μL of α-amylase solution (75 U/mL in the final mixture), and water. Therefore, the ratio of 20 mg dry extract: 1 mL oral phase (200 mg extract:10 mL oral phase) was chosen in accordance with previous studies.^19^ Digestion took place in an orbital incubator (Stuart S150 Orbital Incubator, Barloworld, Espaa) at 37 °C in the darkness and light shaking (2 min for oral phase, 2 h for gastric phase and for intestinal phase). Enzymatic activity was adjusted according to the protocol (75 U/mL of α-amylase, 2000 U/mL of porcine pepsin, 60 U/mL of lipase from *Rhizopus oryzae*, 100 U/mL of porcine pancreatin based on trypsin activity, and 10 mM of bile salts).

An additional assay (pH simulation assay) was performed to determine the influence of the pH conditions on PCs throughout the digestion process. In this case, GID was also developed according to the INFOGEST standardized protocol,^17^ but without adding enzymes and bile salts.

In parallel, GID was performed using similar conditions for the oral and gastric phases, although intestinal digestion took place inside a dialysis bag (Standard RC tubing, MWCO 6–8 kDa, Spectra Pro 1 Dialysis Membrane, Irving, TX, USA). The dialysis bags were immersed in a PBS (phosphate-buffered saline, pH 7.2) bath (500 mL) and incubated at 37 °C and 130 rpm.^20^ After that, the IN fraction (corresponding to nonbioaccessible compounds) was obtained. The difference between intestinal digestion phase and the IN fraction allowed us to calculate the bioaccessibility of PCs as follows:^20^

$$\mathrm{Bioaccessibility}\left(\%\right)=\frac{\left[\mathrm{PCs}\right]intestinal\;phase-\left[PCs\right]IN\;fraction}{\left[\mathrm{PCs}\right]\;\mathrm{ethanolic extracts}}\times 100$$

where [PCs]_intestinal phase_ = phenolic compound concentration after intestinal digestion (mg of phenolic compounds/g of extract).

[PCs]_IN fraction_ = phenolic compound concentration after intestinal digestion into a dialysis bag (mg of phenolic compounds/g of extract).

[PCs]= phenolic compound concentration in the extract before the digestion process (mg of phenolic compounds/g of extract).

Aliquots from the gastric phase, intestinal phase, and IN fraction were freeze-dried and stored at −20 °C in the darkness until further analysis.

### 2.4 *In Vitro* Colonic Fermentation

Three independent fermentation experiments were conducted for the IN fraction of both extracts (yarrow and marjoram) as previously described.^21^ Fecal samples were collected at the Universidad Autónoma de Madrid from three healthy volunteers (age 30 ± 5 years old, 2 males, 1 female) who had not ingested antibiotics or probiotics or prebiotics for at least 6 months prior to the study. All participants provided written informed consent for their participation in the study.

Fecal slurry was obtained by mixing the feces with PBS (0.1 M at pH 7) in a 1:10 ratio (w/v) and homogenized using a Stomacher 400 Circulator (Seward, USA) at maximum speed for 3 min. Then, 100 mL Erlenmeyer flasks were filled with 0.6 g of IN fractions lyophilized, 54 mL of colon nutrient medium at pH 6.8 (peptone water (2 g/L), yeast extract (2 g/L), NaCl (0.1 g/L), K_2_HPO_4_ (0.04 g/L), KH_2_PO_4_ (0.04 g/L), NaHCO_3_ (2 g/L), MgSO_4_·7H_2_O (0.01 g/L), CaCl_2_·6H_2_O (0.01 g/L), Tween 80 (2 mL/L), hemin (0.05 g/L), vitamin K (10 μL/L), l-cysteine (0.5 g/L), bile salts (0.5 g/L), and distilled water), and 6 mL of fecal slurry. In parallel, two control incubations were conducted: a) a negative control without extracts (fecal microbiota and colon nutrient medium) and b) a positive control using inulin (inulin from artichoke, Orafti-GR, Beneo, polymerization degree ≥10), which was previously digested (200 mg) in the same conditions as extracts. All fermentation flasks were incubated for 48 h, at 37 °C, pH 6.8, under anaerobic conditions, and at 120 rpm, simulating the conditions of the distal colon. The mixture was shaken for 1 min at 120 rpm. After that, two aliquots (5 mL) were collected (time: 0 h). Moreover, the aliquots were taken at 24 and 48 h. One of these two aliquots was used for the microbial count, and another was centrifuged at 35060 g, 4 °C, and 10 min. The supernatants were freeze-dried and stored at −20 °C until further analysis of PCs, phenolic metabolites, ammonium, and SCFAs. The pellets were stored at −80 °C for further metataxonomic analysis.

### 2.5 Analysis of Phenolic Compounds and Microbial Phenolic Metabolites

The analysis of phenolic compounds and phenolic microbial catabolites was performed according to Summer et al.^22^ recommendations for metabolite identification and quantification.

In that regard, the identification of the phenolic composition of plant extracts, GID samples and supernatants of the colonic fermentation assays were performed in an Agilent 1290 UHPLC system (Agilent Technologies, Santa Clara, CA, USA) coupled to an ultrahigh-resolution QTOF instrument (6540 UHD, Agilent Technologies, Santa Clara, CA, USA) and in line with a photodiode-array detector (PAD) (G4212A, Agilent Technologies, Santa Clara, CA, USA). The mass spectrometer operated in the negative and positive ion mode using an ESI source (Agilent Jet Stream, AJS, Santa Clara, CA, USA), and the parameters for MS analysis were described by Siles-Sanchez et al.^12^ An ACE Excell 3 Super C18 column (150 × 4.6 mm, 3 μm particle size) at 35 °C and protected by a guard column (10 × 3 mm) was used for chromatographic separation of PCs. Water with 0.1% formic acid (v/v) and acetonitrile with 0.1% formic acid (v/v) were, respectively, employed as solvent A and solvent B, following the gradient conditions proposed by Villalva et al.^19^ Filtered samples (0.22 μm PVDF filter, Symta, Madrid, Spain) dissolved in ethanol were injected. Phenolic compounds were tentatively identified based on their accurate masses and by comparing their fragmentation mass spectra with the NIST MS Data library and UV–vis spectra (Tables S1–S3). Moreover, for quantification purposes, an HPLC 1260 Infinity series system with a PAD (Agilent Technologies Inc., Santa Clara, CA, USA) was used following the methodology mentioned above.^19^ Analytical standards (HPLC purity ≥95%) from different suppliers (Sigma-Aldrich, Phytolab, Extrasynthese S.A, HWI Analytik GMBH, and Fluka) were used to develop individual calibration curves. Exceptions were made for apigenin-hexoside-pentoside and schaftoside isomers, which were quantified using schaftoside standard. Hydroxyluteolin-hexoside and luteolin-hexoside were quantified using luteolin-7-*O*-glucoside, quercetin-hexoside with rutin, caffeoylarbutin isomer with caffeic acid, methylapigenin-hexoside with vitexin, hydroxymethoxyflavone-hexoside with apigenin-7-*O*-glucoside and hydroxyflavone-hexuronide with apigenin-7-*O*-glucuronide. Similarly, the lithospermic acid isomer and the salvianolic acid isomer were quantified using the lithospermic acid and the salvianolic acid standards, respectively. Lastly, hydroxymethoxyflavones were quantified based on the apigenin calibration curve, in accordance with their UV–vis spectrum.

Moreover, the analysis of the phenolic metabolites from *in vitro* colonic fermentation samples was completed by using UPLC-ESI-TQ MS following the methodology reported previously.^23,24^ The liquid chromatographic system was Waters Acquity UPLC (Waters, Milford, USA) equipped with a binary pump, an autosampler conditioned at 10 °C, and a heated column compartment (40 °C). The column employed was BEH-C18, 2.1 × 100 mm, and 1.7 μm particle size from Waters (Milford, USA). Chromatographic and detection conditions, data processing, as well as the MS/MS parameters (cone voltage, collision energy, and MRM transition) of the 19 phenolic compounds targeted in the present study (simple phenols, benzoic acids, phenylacetic acids, phenylpropionic acids and cinnamic acids) were performed as previously reported.^24^ The ESI was operated in negative ionization mode. All metabolites were quantified using the calibration curves of their corresponding standards commercially available from different suppliers (Sigma-Aldrich, St. Louis, USA; Phytolab, Vestenbergsgreuth, Germany; and Extrasynthese, Genay, France). Data acquisition and processing were realized with MassLynx version 4.1 software. The analysis was performed using supernatants of the colonic fermentation assays previously defrosted and filtered through 0.22 μm PVDF filters. All analyses were performed in duplicate for each experiment (*n* = 6).

### 2.6 Analysis of Fat Content

Fat content of marjoram and yarrow extract was determined according to the Folch method.^25^ Results were expressed as g of fat per 100 g of extract (%).

### 2.7 Analysis of Ammonium Content

The proteolytic activity of colonic microbiota was assayed by measuring the production of ammonium ion (NH_4_^+^) in the supernatants from the fermented samples, using the ammonium test (Photometric Spectroquant Ammonium Reagent Test, Merck and Co., Kenilworth, NJ, USA).

### 2.8 Analysis of Short-Chain Fatty Acids

Short-chain fatty acids (SCFA) were determined by SPME-GC-MS according to Cueva et al.^26^ Briefly, 290 μL of *in vitro* colonic fermentation samples or calibration stock solutions was added with 10 μL of an internal standard solution (2-methylvaleric acid) and 30 μL of 0.9 M H_2_SO_4_ solution (pH = 2). One hundred μL of the acidified sample was then transferred to a 20 mL hermetically closed vial. The extraction procedure was automatically performed by using a CombiPAL system (CTC Analytics AG, Zwingen, Switzerland), with a 50/30 μm SPME fiber. SPME conditions included a temperature of 40 °C for 25 min, without salt addition. Desorption was performed in the injector of the GC-MS system (Agilent 7890A, Agilent 5975C MS) in splitless mode for 2 min. The chromatographic separation was performed in a DB-FFAP capillary column (30 m × 0.25 mm i.d. × 0.25 μm film thickness) (J and W, Agilent), with helium as carrier gas at a flow rate of 1 mL/min. The oven temperature was initially held at 100 °C for 5 min, then increased at 5 °C/min to 250 °C, and held for 12 min. The acquisitions were performed in scan (from 35 to 350 amu) and electronic impact mode (70 eV). Other MS conditions were 270, 150, and 230 °C for the transfer line, quadrupole, and ion source, respectively. Compound identification was carried out by comparing retention times and mass spectra with the NIST 2.0 library. Quantitative data were obtained by calculating retention times and mass spectra and by calculating the peak area of each compound relative to the internal standard. Calibration curves for each compound (acetic, propionic, and butyric acids) were generated from a 5000 mg/L stock standard solution.

### 2.9 Microbial Community Analysis

#### 2.9.1 Microbial Plate Counting

Immediately after sampling (0, 24, and 48 h), 10-fold serial dilutions of each colonic sample were plated on different types of media.^27^ Plate counting was done in triplicate, and data were expressed as log (CFU/mL).

#### 2.9.2 DNA Extraction, 16S rDNA Sequencing, and Data Processing

Pellets obtained from colonic fermentation at different times were used for total DNA extraction using the QIAamp Fast DNA Stool Mini Kit (Qiagen, Hilden, Germany) following the manufacturer’s protocol with slight modifications. Extracted DNA was estimated using a NanoDrop 1000 spectrophotometer (NanoDrop Technologies, INC, Rockland, ME, USA) and stored at −20 °C until further analysis. The V3-V4 region of the 16S rRNA gene was amplified, a two-step Illumina PCR protocol was followed to prepare the libraries, and samples were submitted to 2 × 300 bp paired-end sequencing (Illumina MiSeq instrument, Illumina, San Diego, CA, USA). Universal primers S-D-Bact0341-*b*-S-17 (CCTACGGGNGGCWGCAC) and S-D-Bact-129 0785-a-A-21 (GACTACHVGGGTATCTAATCC) were used for marker amplification. Bioinformatic analysis for fastq files containing raw reads was performed under an RStudio v.1.3.1093 environment (https://www.rstudio.com/), following pipeline and methods previously described.^28^ Alpha-biodiversity metrics were estimated using the amplicon sequence variants (ASVs) through the “Phyloseq” package.^29^ Beta-diversity was evaluated employing the Bray–Curtis dissimilarity matrix represented by nonmetric multidimensional scaling (NMDS).

### 2.10 Statistical Analysis

The results shown represent mean ± standard deviation of at least three replicates obtained in each experiment. Data from PCs, phenolic metabolites, microbial plate counting, SCFAs, and ammonium ion were analyzed using one-way ANOVA and Tukey’s b post hoc test to determine statistical differences between samples (*p*-value < 0.05) (Statistics 26 Core System, SPSS Inc., IBM Company, New York, NY, USA). A two-way ANOVA test was used to study the differences between fermentation samples at different incubation times for 16S rRNA gene-based sequencing analysis (alpha-diversity metrics and relative abundance of microbial taxa). Least significant differences were calculated with the Games-Howell test using the XLSTAT Statistic for Microsoft Excel, version 2020.1 (Addinsoft SARL., Paris, France).

## 3 Results and Discussion

### 3.1 Influence of Gastrointestinal Digestion on Phenolic Compounds of Yarrow and Marjoram Extracts

The phenolic profile of yarrow extract (YE) (Table 1) showed 3,5-dicaffeoylquinic acid (DCQA), methylapigenin-hexoside, vicenin II, luteolin-7-*O*-glucoside, and schaftoside acid as the main components of the 32 quantified PCs. For marjoram extract (ME) (Table 2), 22 PCs were identified, highlighting the presence of arbutin and rosmarinic acid, 47% and 33% of total PCs, respectively. This phenolic composition was in accordance with previous studies for both extracts.^30–32^

**Table 1:** Phenolic Compounds Content of Yarrow Extract (YE) and Gastrointestinal Digestion Phases (Gastric Phase, Intestinal Phase, and IN Fraction), Bioaccessibility, and Phenolic Compounds Content in the Fermentation Samples[Table-fn tbl1fn2]

		Gastrointestinal digestion samples				Fermentation samples		
	mg/g dry extract %					mg/L fermentation medium		
	YE	Gastric phase	Intestinal phase	IN fraction	Bioaccesibility	0 h	24 h	48 h
**Phenolic acids**								
**Hydroxycinnamic acids**								
Neochlorogenic acid	0.26 ± 0.01^d^	0.17 ± 0.00^b^	0.20 ± 0.01^c^	0.15 ± 0.01^a^	19	n.d	0.48 ± 0.09	n.d
Chlorogenic acid	2.68 ± 0.02^c^	2.32 ± 0.03^b^	2.53 ± 0.08^c^	1.62 ± 0.14^a^	34	0.78 ± 0.13	n.d	n.d
Cryptochlorogenic acid	0.06 ± 0.00^a^	0.06 ± 0.00^a^	0.15 ± 0.01^c^	0.09 ± 0.01^b^	100	0.04 ± 0.01	n.d	n.d
Caffeic acid	0.21 ± 0.01^c^	0.14 ± 0.01^b^	0.20 ± 0.02^c^	0.11 ± 0.01^a^	43	15.64 ± 0.38^c^	0.13 ± 0.01^b^	0.09 ± 0.01^a^
3,4-Dicaffeoylquinic acid	0.94 ± 0.01^c^	0.55 ± 0.00^a^	1.03 ± 0.02^d^	0.81 ± 0.08^b^	23	1.87 ± 0.06	n.d	n.d
1,5-Dicaffeoylquinic acid	0.81 ± 0.01^d^	0.43 ± 0.01^a^	0.55 ± 0.03^bc^	0.51 ± 0.02^b^	5	0.44 ± 0.02	n.d	n.d
3,5-Dicaffeoylquinic acid	10.97 ± 0.12^c^	6.50 ± 0.10^a^	8.56 ± 0.37^b^	6.40 ± 0.71^a^	20	1.23 ± 0.03	n.d	n.d
4,5-Dicaffeoylquinic acid	1.91 ± 0.02^b^	1.36 ± 0.01^a^	1.95 ± 0.01^c^	1.28 ± 0.11^a^	35	0.31 ± 0.02	n.d	n.d
**Flavonoids**								
**Flavones**								
Vicenin II	5.83 ± 0.06^d^	3.93 ± 0.03^b^	4.40 ± 0.14^c^	2.91 ± 0.23^a^	26	9.86 ± 0.44^c^	8.65 ± 0.37^b^	7.93 ± 0.44 ^a^
Schaftoside acid isomer I	1.19 ± 0.08^c^	0.86 ± 0.00^b^	0.94 ± 0.02^b^	0.61 ± 0.05^a^	28	1.97 ± 0.05^b^	1.87 ± 0.12^b^	1.63 ± 0.09^a^
Schaftoside acid isomer II	2.94 ± 0.05^c^	1.99 ± 0.07^b^	2.18 ± 0.07^b^	1.46 ± 0.21^a^	24	6.18 ± 0.22^c^	5.23 ± 0.37^b^	3.71 ± 0.23^a^
Schaftoside acid	5.12 ± 0.09^d^	3.52 ± 0.05^b^	4.03 ± 0.14^c^	2.58 ± 0.22^a^	28	7.62 ± 0.45^a^	6.92 ± 0.50^a^	6.96 ± 0.07^a^
Homoorientin	0.47 ± 0.01^c^	0.35 ± 0.01^b^	0.36 ± 0.01^b^	0.24 ± 0.02^a^	26	0.36 ± 0.02^b^	0.28 ± 0.01^a^	0.27 ± 0.03^a^
Apigenin-hexoside-pentoside	2.92 ± 0.03^d^	1.99 ± 0.04^b^	2.26 ± 0.08^c^	1.54 ± 0.13^a^	25	4.90 ± 0.19^c^	4.42 ± 0.26^b^	4.14 ± 0.23^a^
Luteolin-hexoside	3.37 ± 0.04^d^	2.12 ± 0.02^b^	2.44 ± 0.08^c^	1.46 ± 0.12^a^	29	3.03 ± 0.13^b^	2.41 ± 0.11^a^	2.33 ± 0.12^a^
Hydroxyluteolin-hexoside	2.64 ± 0.03^c^	1.35 ± 0.01^b^	1.35 ± 0.05^b^	1.28 ± 0.04^a^	3	0.08 ± 0.01^b^	0.06 ± 0.02^a^	0.02 ± 0.01^a^
Vitexin	1.02 ± 0.01^c^	0.52 ± 0.00^a^	0.61 ± 0.01^b^	0.57 ± 0.06^ab^	4	1.35 ± 0.05^b^	1.44 ± 0.06^b^	1.29 ± 0.07^a^
Methylapigenin-hexoside	6.11 ± 0.06^d^	3.76 ± 0.00^b^	4.47 ± 0.18^c^	3.10 ± 0.24^a^	22	9.28 ± 0.42^b^	7.42 ± 0.51^a^	6.97 ± 0.02^a^
Luteolin-7-*O*-glucoside	5.48 ± 0.05^d^	3.49 ± 0.00^b^	4.00 ± 0.15^c^	2.65 ± 0.22^a^	25	n.d	n.d	n.d
Hydroxydimethoxyflavone-hexoside	0.76 ± 0.01^c^	0.49 ± 0.00^b^	0.54 ± 0.05^b^	0.40 ± 0.03^a^	18	1.78 ± 0.05^c^	1.52 ± 0.08^b^	0.95 ± 0.07^a^
Apigenin-7-*O*-glucoside	1.14 ± 0.03^c^	0.66 ± 0.02^b^	0.71 ± 0.02^b^	0.50 ± 0.06^a^	18	n.d	n.d	n.d
Trihydroxyflavone-hexuronide	0.93 ± 0.05^c^	0.37 ± 0.03^a^	0.65 ± 0.10^b^	0.43 ± 0.05^a^	24	n.d	n.d	n.d
Luteolin	1.28 ± 0.01^d^	0.65 ± 0.02^b^	0.84 ± 0.05^c^	0.43 ± 0.04^a^	32	2.63 ± 0.33	n.d	n.d
Apigenin	0.38 ± 0.00^d^	0.19 ± 0.01^b^	0.27 ± 0.02^c^	0.15 ± 0.01^a^	32	0.64 ± 0.13^b^	0.03 ± 0.00^a^	n.d
Diosmetin	0.45 ± 0.00^d^	0.24 ± 0.01^b^	0.30 ± 0.02^c^	0.19 ± 0.01^a^	24	0.85 ± 0.22^c^	0.36 ± 0.07^b^	0.25 ± 0.01^a^
Dihydroxydimethoxyflavone	2.03 ± 0.03^c^	0.83 ± 0.03^a^	1.17 ± 0.10^b^	1.13 ± 0.07^b^	2	1.41 ± 0.22^b^	0.41 ± 0.05^a^	0.34 ± 0.03^a^
Dihydroxytrimethoxyflavone	2.76 ± 0.11^c^	1.18 ± 0.05^a^	1.65 ± 0.13^b^	1.50 ± 0.07^b^	5	1.85 ± 0.28^b^	0.69 ± 0.05^a^	0.59 ± 0.06^a^
Hydroxytetramethoxyflavone	4.60 ± 0.05^c^	2.18 ± 0.13^a^	3.18 ± 0.26^b^	3.05 ± 0.37^b^	3	2.73 ± 0.13^c^	0.77 ± 0.08^b^	0.65 ± 0.07^a^
Hydroxytrimethoxyflavone	1.68 ± 0.02^c^	0.64 ± 0.05^a^	1.03 ± 0.02^b^	1.05 ± 0.09^b^	0	0.56 ± 0.18	n.d	n.d
**Flavonols**								
Quercetin-hexoside	0.99 ± 0.10^c^	0.53 ± 0.03^a^	0.73 ± 0.07^b^	0.49 ± 0.02^a^	24	0.02 ± 0.01	n.d	n.d
Rutin	1.51 ± 0.02^d^	1.18 ± 0.01^b^	1.31 ± 0.04^c^	1.06 ± 0.09^a^	16	0.32 ± 0.07	n.d	n.d
Quercetin	0.26 ± 0.00^b^	0.15 ± 0.01^a^	0.18 ± 0.02^b^	0.15 ± 0.01^a^	12	0.44 ± 0.06	n.d	n.d
**Flavanones**								
Eriodyctiol	-	-	-	-	-	0.09 ± 0.00^a^	0.56 ± 0.08^b^	0.63 ± 0.10^b^
Naringenin	-	-	-	-	-	0.13 ± 0.00	n.d	n.d
**Flavanonols**	-	-	-	-	-			
Taxifolin	-	-	-	-	-	0.43 ± 0.05	0.55 ± 0.07	0.49 ± 0.02
**Σ Phenolic compounds**	**73.70**	**44.70**	**54.77**	**39.9**	**20**	**78.82**	**44.20**	**39.24**

I Data are expressed as mean ± standard deviation (*n* = 6). Superscript letters mean statistically significant differences (*p*-value *<* 0.05) between extract and gastrointestinal digestion phases (gastric phase, intestinal phase, and IN fraction) or between fermentation samples (0 h, 24 h, 48 h). n.d. (not detected).

**Table 2:** Phenolic Compounds of Marjoram Extract (ME) and Gastrointestinal Phases (Gastric Phase, Intestinal Phase, and IN Fraction), Bioaccessibility, and Phenolic Compounds Content in the Fermentation Samples[Table-fn tbl2fn2]

		Gastrointestinal digestion samples				Fermentation samples		
	mg/g dry extract %					mg/L fermentation medium		
	ME	Gastric phase	Intestinal phase	IN fraction	Bioaccesibility	0 h	24 h	48 h
**Simple phenols**								
Arbutin	67.98 ± 1.13^c^	57.21 ± 2.37^b^	58.14 ± 0.09^b^	23.23 ± 2.06^a^	51	75.57 ± 6.62	n.d.	n.d.
Hydroquinone	n.d.	0.73 ± 0.05^b^	0.77 ± 0.01^b^	0.10 ± 0.01^a^	-	6.04 ± 0.52^a^	14.47 ± 1.07^c^	12.36 ± 0.50^b^
**Phenolic acids**								
**Hydroxycinnamic acids**								
Caffeic acid	0.43 ± 0.01^c^	0.32 ± 0.02^b^	0.35 ± 0.01^b^	0.16 ± 0.01^a^	44	15.73 ± 0.40^b^	0.15 ± 0.01^a^	0.12 ± 0.00^a^
Caffeoylarbutin isomer	2.26 ± 0.04^c^	1.69 ± 0.08^b^	1.63 ± 0.01^b^	0.93 ± 0.11^a^	31	n.d.	n.d.	n.d.
Rosmarinic acid	38.12 ± 0.76^d^	34.53 ± 0.72^c^	27.96 ± 0.43^b^	15.41 ± 0.21^a^	33	4.97 ± 0.53	n.d.	n.d.
Lithospermic acid isomer	7.21 ± 0.03^d^	6.60 ± 0.13^c^	3.82 ± 0.82^b^	1.88 ± 0.24^a^	27	1.71 ± 0.12^b^	1.32 ± 0.03^b^	1.08 ± 0.10^a^
Salvianolic acid isomer	1.87 ± 0.01^c^	1.58 ± 0.05^c^	1.48 ± 0.14^b^	0.79 ± 0.08^a^	37	1.47 ± 0.11^b^	1.49 ± 0.05^b^	1.34 ± 0.03^a^
**Flavonoids**								
**Flavones**								
Vicenin II	4.49 ± 0.03^c^	3.94 ± 0.01^b^	4.00 ± 0.20^b^	1.65 ± 0.20^a^	52	4.46 ± 0.31^b^	4.22 ± 0.29^ab^	3.83 ± 0.47^a^
Homoorientin	0.40 ± 0.01^b^	0.44 ± 0.04^b^	0.41 ± 0.01^b^	0.23 ± 0.03^a^	45	n.d.	n.d.	n.d.
Orientin	0.62 ± 0.01^c^	0.60 ± 0.02^b^	0.59 ± 0.01^b^	0.26 ± 0.06^a^	53	0.44 ± 0.01^c^	0.28 ± 0.0^b^	0.01 ± 0.00^a^
Luteolin-7-*O*-glucoside	1.11 ± 0.02^c^	0.99 ± 0.03^c^	0.83 ± 0.01^b^	0.40 ± 0.03^a^	39	n.d.	n.d.	n.d.
Luteolin-7-*O*-glucuronide	3.90 ± 0.01^b^	3.62 ± 0.18^b^	3.37 ± 0.34^b^	0.84 ± 0.14^a^	65	n.d.	n.d.	n.d.
Apigenin-7-*O*-glucuronide	2.80 ± 0.05^b^	2.54 ± 0.04^b^	2.52 ± 0.27^b^	0.88 ± 0.10^a^	59	n.d.	n.d.	n.d.
Luteolin	0.15 ± 0.00^c^	0.15 ± 0.02^b^	0.15 ± 0.00^b^	0.09 ± 0.00^a^	40	1.14 ± 0.01	n.d.	n.d.
Trihydroxymethoxyflavone	1.02 ± 0.02^d^	0.93 ± 0.05^c^	0.13 ± 0.00^b^	0.09 ± 0.01^a^	4	n.d.	n.d.	n.d.
Trihydroxydimethoxyflavone I	2.67 ± 0.05^c^	2.20 ± 0.16^b^	2.08 ± 0.02^b^	1.71 ± 0.15^a^	14	1.98 ± 0.07^c^	0.42 ± 0.07^b^	0.37 ± 0.01^a^
Trihydroxydimethoxyflavone II	0.75 ± 0.01^c^	0.54 ± 0.04^b^	0.15 ± 0.03^a^	0.13 ± 0.01^a^	3	0.30 ± 0.01^b^	0.18 ± 0.04^a^	0.21 ± 0.02^a^
Apigenin	0.04 ± 0.00^b^	0.04 ± 0.00^b^	0.04 ± 0.00^b^	0.02 ± 0.00^a^	50	0.49 ± 0.00	n.d.	n.d.
Trihydroxytrimethoxyflavone	3.54 ± 0.08^c^	2.99 ± 0.23^b^	2.03 ± 0.19^a^	1.94 ± 0.28^a^	3	2.20 ± 0.15^b^	0.32 ± 0.01^a^	0.32 ± 0.01^a^
**Flavanones**								
Eriodyctiol	1.06 ± 0.02^c^	0.96 ± 0.07^b^	0.90 ± 0.01^b^	0.60 ± 0.05^a^	28	0.74 ± 0.05^b^	n.d.	n.d.
Naringenin	0.48 ± 0.01^c^	0.39 ± 0.04^b^	0.37 ± 0.01^b^	0.25 ± 0.00^a^	25	0.83 ± 0.01	n.d.	n.d.
Sterubin	3.07 ± 0.06^c^	2.69 ± 0.31^b^	2.41 ± 0.04^ab^	2.31 ± 0.14^a^	3	3.61 ± 0.14^a^	3.93 ± 0.24^a^	3.68 ± 0.14^a^
**Flavanonols**								
Taxifolin	2.27 ± 0.03^d^	1.96 ± 0.14^c^	1.39 ± 0.06^b^	1.04 ± 0.09^a^	15	0.87 ± 0.02	n.d.	n.d.
**Σ Phenolic compounds**	**146.24**	**127.64**	**115.52**	**54.94**	**41**	**122.55**	**26.78**	**23.32**

I Data are expressed as mean ± standard deviations (*n* = 6). Lowercase letters mean statistically significant differences (*p*-value < 0.05) between extract and gastrointestinal digestion phases (gastric phase, intestinal phase, and IN fraction) or between fermentation samples (0 h, 24 h, 48 h). n.d. (not detected).

The effect of *in vitro* gastric and intestinal digestion on the PCs of YE and ME was evaluated. Oral phase aliquots were not taken as no remarkable modifications of PCs in this phase were reported in literature.^7,33^ Moreover, both extracts were subjected to a pH simulation assay that consisted of the same *in vitro* GID, but without the addition of any digestive enzymes or bile salts. This assay would allow us to evaluate the degradation rate of PCs associated with pH conditions.

In general, the solubilization, isomerization, or degradation of PCs in the digestion medium were dependent on the GID phase and extract studied. In the case of YE, the concentration of most PCs increased after the intestinal phase compared with the gastric phase. The generally low rates of PC loss after the pH simulation assay corroborated the solubilization effect on PCs throughout the digestion process, rather than a degradation (Table S4).

The intestinal phase showed 12–19% more of the main flavones and a higher content of all hydroxycinnamic acids compared to the gastric phase. Hydrolysis of the high fat content of yarrow extract (58.2 ± 2.3%) due to intestinal lipase could be related to the higher solubilization of the PCs in the intestinal phase. In this respect, the PC content after the intestinal phase in the pH simulation test was generally lower than that obtained after the digestion process. This result could confirm the involvement of intestinal lipase in lipid hydrolysis, enhancing the solubilization of phenolic compounds in YE.

In addition, the statistically higher content of 3,4-DCQA, 4,5-DCQA, and cryptochlorogenic acid in the intestinal phase compared to the initial extract might be attributed to an isomerization and/or transformation process from 3,5-DCQA, 1,5-DCQA, and chlorogenic acid, favored by the alkaline pH as has been previously reported.^8,34,35^

On the other hand, ME showed a high solubility after the gastric phase. The two main compounds, arbutin and rosmarinic acid, showed a content of 84% and 91% in the gastric phase compared to the initial extract. This result agreed with those corresponding to the pH simulation assay (Table S5).

However, intestinal digestion caused a loss of specific PCs, such as lithospermic acid, salvianolic acid, rosmarinic acid, and some hydroxymethoxyflavones (trihydroxydimethoxyflavone, trihydroxydimethoxyflavone II, and trihydroxytrimethoxyflavone). This could be associated with the transition from gastric to intestinal pH or their binding to bile salts, with subsequent precipitation, as it has been previously reported in other matrices.^36,37^ In turn, there were no statistical differences between gastric and intestinal phases in the concentration of some specific compounds, e.g., arbutin, caffeoylarbutin isomer, or apigenin-7-*O*-glucuronide, denoting a possible influence of the structure on the degradation rate of compounds during the intestinal phase. Moreover, the slight increase in the caffeic acid content after the intestinal phase could be derived from rosmarinic acid. In addition, hydroquinone should be associated with the deglycosylation of arbutin by digestion enzymes.^38^

In general, a low effect of enzymes and bile salts was observed on PC reduction, as can be seen from the comparison of the phenolic compound content in the samples from the digestion with those from the pH simulation (Tables 1 and 2 vs Tables S4 and S5).

In parallel, an assay of intestinal digestion was performed inside dialysis bags to obtain nonbioaccessible PCs for the colonic *in vitro* fermentation. The use of these membranes has been considered an effective approach to simulate *in vivo* conditions of compound absorption.^39^ Some of the PCs were able to pass through the dialysis membrane, as evidenced by the lower content found in the IN fraction compared to the intestinal phase. Thus, the IN fraction of YE and ME retained 80% and 59% of the total PCs found in the intestinal phase, representing those potentially colon available compounds (nonbioaccessible fraction). These results agree with those reported for other plant extracts or pure phenolic compounds.^40–42^

Furthermore, for YE, the DCQAs showed lower bioaccessibility than monocaffeoylquinic acids. As an example, a bioaccessibility of 34% was estimated for chlorogenic acid, decreasing to 20% for 3,5-DCQA. Moreover, the main flavones showed similar bioaccessibilities (22–29%).

In general, individual PCs of ME showed higher bioaccessibilities, e.g., for arbutin (51%), luteolin-7-*O*-glucuronide (75%), and rosmarinic acid (33%). In line with our results, other authors have pointed out bioaccessibilities of about 40% for a standard of rosmarinic acid, or a rosemary extract,^43^ 4% for 3,5-DCQA in white mugwort extract,^44^ 14% for chlorogenic acid and its isomers from apple and coffee,^45,46^ and 36–58% for luteolin-hexoside and glucuronide derivatives of *Origanum vulgare*, *Melissa officinalis*, and *Lavandula latifolia*.^41^

### 3.2 Colonic Metabolism of Yarrow and Marjoram Phenolic Compounds

The colonic metabolism of those nonbioaccessible PCs of YE and ME, including the phenolic metabolites produced, is shown in Tables 1– [Table tbl3]. To consider exclusively the metabolites produced by microbial catabolism from both plant extracts, a negative control (fecal slurry without extract) and a positive control (fecal slurry with digested inulin) were also incubated.

**Table 3:** Phenolic Metabolites Produced during the *In Vitro* Colonic Fermentation of Yarrow Extract (YE) and Marjoram Extract (ME) and Positive and Negative Controls (mg Compound/L)

	Time (h)	YE	ME	Positive control	Negative control
**Simple phenols**					
Phloroglucinol	0	n.d	n.d	n.d	n.d
	24	2.87 ± 0.20[Table-fn tbl3fn1] ^b^_A_	0.98 ± 0.12^a^_B_	n.d	n.d
	48	3.49 ± 0.42^b^_B_	0.80 ± 0.05^a^_A_	n.d	n.d
Catechol	0	n.d	n.d	n.d	n.d
	24	n.d	1.48 ± 0.19_A_	n.d	n.d
	48	0.40 ± 0.03^a^	2.36 ± 0.16^b^_B_	n.d	n.d
Hippuric acid	0	0.08 ± 0.00^a^_B_	0.10 ± 0.01^b^_B_	0.13 ± 0.02^b^_B_	0.11 ± 0.02^b^_B_
	24	0.08 ± 0.01^d^_B_	0.03 ± 0.00^b^_A_	0.07 ± 0.01^c^_A_	0.02 ± 0.01^a^_A_
	48	0.03 ± 0.00_A_	n.d	n.d	n.d
**Cinnamic acids**					
*p*-Coumaric acid	0	0.24 ± 0.01^a^_B_	0.52 ± 0.04^b^_B_	n.d	n.d
	24	n.d	0.01 ± 0.00_A_	n.d	n.d
	48	0.03 ± 0.00^b^_A_	0.01 ± 0.00^a^_A_	n.d	n.d
Ferulic acid	0	0.85 ± 0.05^a^	1.85 ± 0.20^b^	n.d	n.d
	24	n.d	n.d	n.d	n.d
	48	n.d	n.d	n.d	n.d
**Phenylpropionic acids**					
3-(3-Hydroxyphenyl)-propionic acid	0	0.03 ± 0.00^a^_A_	0.05 ± 0.01^b^_A_	0.06 ± 0.00^c^_A_	0.06 ± 0.00^c^_A_
	24	0.07 ± 0.01^ab^_B_	0.13 ± 0.01^c^_B_	0.06 ± 0.01^a^_A_	0.08 ± 0.00^b^_C_
	48	0.11 ± 0.01^b^_C_	0.16 ± 0.01^c^_C_	0.07 ± 0.00^a^_A_	0.07 ± 0.01^a^_A_
3-(4-Hydroxyphenyl)-propionic acid	0	n.d	n.d	n.d	n.d
	24	2.51 ± 0.10^a^_A_	5.05 ± 0.62^b^_A_	n.d	n.d
	48	3.79 ± 0.51^a^_B_	4.58 ± 0.51^b^_A_	n.d	n.d
**Phenylacetic acids**					
3,4-Dihydroxyphenyl-acetic acid	0	n.d	0.15 ± 0.02_A_	n.d	n.d
	24	n.d	0.24 ± 0.02_B_	n.d	n.d
	48	n.d	0.28 ± 0.03_C_	n.d	n.d
3,4-Dimethoxyphenyl-acetic acid	0	0.19 ± 0.29^a^_A_	n.d	0.03 ± 0.00^a^_A_	0.03 ± 0.00^a^_A_
	24	3.47 ± 0.19^b^_B_	4.24 ± 0.46^c^_A_	0.10 ± 0.01^a^_B_	0.14 ± 0.02^a^_B_
	48	3.65 ± 0.23^b^_B_	4.73 ± 0.41^c^_B_	0.15 ± 0.02^a^_C_	0.16 ± 0.03^a^_B_
4-Hydroxyphenyl-acetic acid	0	0.58 ± 0.07^a^_A_	0.60 ± 0.06^a^_A_	0.87 ± 0.10^b^_A_	0.85 ± 0.07^b^
	24	5.43 ± 0.41^b^_B_	10.26 ± 0.60^d^_B_	8.97 ± 0.75^c^_B_	3.39 ± 0.22^a^
	48	0.48 ± 0.06^a^_A_	12.97 ± 0.81^c^_C_	9.44 ± 0.62^b^_B_	0.44 ± 0.03^a^
3-Hydroxyphenyl-acetic acid	0	n.d	0.10 ± 0.05^b^_B_	0.07 ± 0.01^ab^_B_	0.04 ± 0.01^a^_A_
	24	n.d	0.04 ± 0.01^a^_A_	0.07 ± 0.01^b^_B_	0.10 ± 0.01^c^_B_
	48	0.08 ± 0.01^b^	0.10 ± 0.02^b^_B_	0.04 ± 0.00^a^_A_	0.09 ± 0.01^b^_B_
Phenylacetic acid	0	1.65 ± 0.12^a^_A_	1.59 ± 0.08^a^_A_	3.12 ± 0.50^b^_A_	1.81 ± 0.25^a^_A_
	24	8.99 ± 1.07^b^_B_	9.11 ± 0.69^b^_B_	6.63 ± 0.60^a^_B_	12.51 ± 0.51^c^_B_
	48	11.20 ± 1.40^b^_C_	12.08 ± 2.21^b^_C_	6.50 ± 0.42^a^_B_	29.09 ± 1.00^c^_C_
**Benzoic acids**					
Gallic acid	0	0.04 ± 0.01_A_	n.d	n.d	n.d
	24	0.11 ± 0.02^b^_C_	0.05 ± 0.01^a^	n.d	n.d
	48	0.08 ± 0.01_B_	n.d	n.d	n.d
Protocatechuic acid	0	0.72 ± 0.05^b^_C_	0.10 ± 0.01^a^_B_	n.d	n.d
	24	0.61 ± 0.05^c^_B_	0.13 ± 0.02^b^_C_	0.05 ± 0.01^a^_B_	0.04 ± 0.01^a^_A_
	48	0.13 ± 0.02^c^_A_	0.08 ± 0.01^b^_A_	0.03 ± 0.00^a^_A_	0.04 ± 0.00^a^_A_
Vanillic acid	0	0.60 ± 0.06^a^_A_	1.54 ± 0.08^bA^_B_	n.d	n.d
	24	0.81 ± 0.09^a^_B_	1.65 ± 0.16^b^_B_	n.d	n.d
	48	0.90 ± 0.12^a^_B_	1.39 ± 0.12^b^_A_	n.d	n.d
Syringic acid	0	n.d	0.62 ± 0.04_A_	n.d	n.d
	24	0.23 ± 0.04^a^_A_	0.86 ± 0.18^b^_B_	n.d	n.d
	48	0.37 ± 0.04^a^_B_	0.84 ± 0.05^b^_B_	n.d	n.d
3-*O*-Methylgallic acid	0	n.d	n.d	n.d	n.d
	24	n.d	0.03 ± 0.00_B_	n.d	n.d
	48	n.d	0.02 ± 0.00_A_	n.d	n.d
Salicylic acid	0	0.14 ± 0.02^c^_A_	0.05 ± 0.00^b^_A_	0.02 ± 0.00^a^_A_	0.02 ± 0.00^a^_A_
	24	0.36 ± 0.03^c^_B_	0.08 ± 0.01^b^_B_	0.02 ± 0.00^a^_A_	0.02 ± 0.00^a^_A_
	48	0.40 ± 0.03^c^_C_	0.09 ± 0.01^b^_B_	0.04 ± 0.00^a^_B_	0.02 ± 0.00^a^_A_
4-Hydroxybenzoic acid	0	0.99 ± 0.09^b^_A_	6.69 ± 0.30^c^_C_	0.25 ± 0.05^a^_A_	0.29 ± 0.01^a^_A_
	24	1.70 ± 0.07^b^_C_	5.88 ± 0.33^c^_B_	0.64 ± 0.03^a^_C_	0.51 ± 0.08^a^_B_
	48	1.60 ± 0.07^c^_B_	3.31 ± 0.18^d^_A_	0.55 ± 0.01^a^_B_	0.91 ± 0.06^b^_C_

I Data are expressed as mean ± standard deviation (*n* = 6). Lowercase letters mean statistically significant differences (*p*-value < 0.05) between samples at the same time point (0 h, 24 h, 48 h). Uppercase letters denote statistically significant differences (*p*-value < 0.05) between time points within each sample. n.d. (not detected).

As expected, most of the parent phenolic compounds of both extracts were detected at the beginning of the assay. However, some of them were quickly metabolized even in the time interval to prepare samples for chromatographic analysis (e.g., luteolin-7-*O*-glucoside).

Moreover, the evolution of PCs at 24 and 48 h was dependent on the phenolic structure of each compound. Most of the phenolic acids, as well as *O*-glycosidic flavonoids or their aglycone forms (e.g., luteolin-7-*O*-glucoside, rutin, luteolin, apigenin, etc.), were quickly metabolized by the fecal microbiota. On the other hand, *C*-glycosylated and methoxylated derivatives of flavonoids (e.g., vicenin II, vitexin, sterubin, etc.) suffered limited losses during the assay. These results agree with other studies^47^ and with the higher strength of the C–C bond compared to the C–O bond. Furthermore, small quantities of taxifolin, naringenin, and eriodyctiol were also found in fermentation samples of yarrow at 0 h (Table 1), possibly because of a hydrogenation process from quercetin, apigenin, and luteolin.^48,49^ For ME, hydroquinone stood out as a fermentation product of arbutin.^38^ Several authors have pointed out that the bioconversion of PCs by gut microbiota is highly variable because of their chemical structure, the ability of microbiota to produce different enzymes such as β-glucuronidases or α-glucosidases needed for PC catabolism, and the composition of microbial communities.^50,51^ Therefore, the influence of donor microbiota on the metabolism of these compounds cannot be ruled out. In fact, Vollmer et al.^47^ noted that the corresponding conversion rate of vitexin (*C*-glycosidic derivative of apigenin) was time and fecal donor-dependent.

In parallel to this parent microbial degradation, the formation of another 19 PC metabolites from microbial fermentation was noticed (Table 3).

In that regard, several benzoic acids were identified. The presence of gallic acid in yarrow samples would be related to the microbial metabolism of syringic acid by demethylation.^52,53^ In addition, 4-hydroxybenzoic acid, or even simple phenols such as catechol, should be proposed as metabolic products of CQAs and/or flavonoids.^48,54^ Thus, 4-hydroxyphenylpropionic acid or 4-hydroxyphenylacetic acid would act as intermediate metabolites of 4-hydroxybenzoic acid production.^55^ Moreover, an alternative route could contribute to the appearance of benzoic acids such as vanillic acid, syringic acid, or catechol in colonic fermentation samples. In this sense, these compounds could also be generated from hydroxy- or hydroxymethoxyflavones, where hydroxy- or hydroxymethoxyphenylpropionic acid would be intermediate metabolites. For instance, syringic acid could be the metabolic product of C-ring cleavage of a hypothetical hydroxymethoxyflavone, such as tricin. Moreover, the noticeably higher content of catechol found in ME compared to YE should be a degradation product of arbutin. This would involve the generation of a hydroquinone moiety from arbutin,^56^ its subsequent dehydroxylation, and later hydroxylation to yield catechol. This pathway agrees with other reported conversions.^57^

The metabolism of flavonoids from the digested extracts should also be represented by the identification of other metabolites such as phloroglucinol, whose content stood out at 24 and 48 h. This metabolite has been identified in the degradation routes of quercetin, luteolin, or apigenin,^58^ whose content was especially significant in the YE. Another remarkable compound identified was the 3-(4-hydroxyphenyl)-propionic acid, which has been described as the metabolite of apigenin, luteolin, their derivatives, and hydroxymethoxyflavones, but also as the metabolic product of naringenin.^47,58,59^

Phenolic acid metabolism could also be reflected by the presence of caffeic acid (Tables 1 and 2) from CQAs (YE) or rosmarinic acid (ME), albeit this compound was also identified in the initial plant extract.^48,60^ Moreover, the presence of coumaric acid could be related to the further dehydroxylation of caffeic as has been previously described.^54,55,61^ Furthermore, the metabolite 3,4-dihydroxyphenylacetic acid, only identified in ME samples, could also come from rosmarinic acid since it has been described in the catabolic pathways of caffeic acid derivatives.^55,61^ Following the phenylacetic acid family, the compound 4-hydroxyphenylacetic acid has been reported as a common metabolite in the degradation pathways of both hydroxycinnamic acids and flavonoids.^47,48^ 3,4-Dimethoxyphenyl acetic acid also emerged as one of the main metabolites of both extracts. This compound could come from the metabolism of methoxyflavones, although its formation from the methoxylation of other types of flavonoids has not been ruled out.

Hippuric acid was detected in all samples, including the controls. It is known that this compound increases with the intake of phenolic compounds and protein. Therefore, its presence in all samples would be associated with the volunteers’ usual diet. Nevertheless, this compound was only present in yarrow samples at 48 h, which could indicate its formation from the degradation of quinic acid as other studies have suggested.^48,62^

Finally, no differences were found in any sample for 3-hydroxyphenylacetic acid, while a higher phenylacetic acid content was observed in the negative control. On the other hand, the positive control showed a higher content of 4-hydroxyphenylacetic acid than the negative one or YE, although lower than ME. Therefore, no clear effect could be attributed to any of the studied extracts. In that regard, the presence of these metabolites could be more likely due to basal fecal metabolism. Nevertheless, the partial influence of the PCs from yarrow and marjoram digested extracts on the content of these two metabolites cannot be ruled out.

So, it can be concluded that most of the identified metabolites are derived from the specific microbial metabolism of PCs from both extracts. Figure 1 summarizes the proposed metabolic pathway for colonic fermentation of yarrow and marjoram extract.

**Figure 1:**
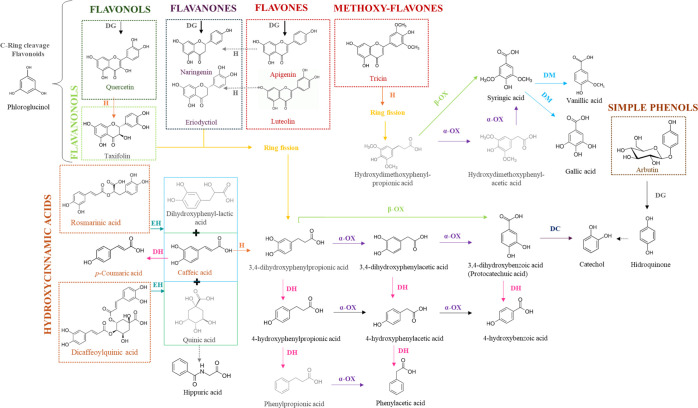
Proposed metabolic pathways of yarrow and marjoram phenolic compounds by colon microbiota. Black compounds are those identified in the fermentative samples, and gray compounds are those not identified. α-OX (α-oxidation), β-OX (β-oxidation), DC (decarboxylation), DG (deglycosylation), DH (dehydroxylation), DM (demethylation), EH (ester hydrolysis), H (hydrogenation), M (methylation).

The generation of these phenolic metabolites could indeed be involved in the *in vivo* biological activities of these plant extracts. For example, the compound 3-(4-hydroxyphenyl)-propionic acid has been evaluated for its protective effect against oxidative damage.^63^ The compound 3,4-dihydroxyphenylacetic acid, found exclusively in marjoram samples, has been shown to be more effective in certain biological activities than its parent PCs. Some of the demonstrated properties have been related to its antiproliferative, antioxidant, and anti-inflammatory activities.^64^ Also, phloroglucinol has been evaluated for its potential as antioxidant,^58^ as well as caffeic acid which could act as an antitumor agent or as a neuroprotector, among other bioactivities.^65^

### 3.3 Influence of Yarrow and Marjoram Extracts on Ammonium and SCFA Content during Colonic Fermentation

Ammonium ion content was measured during the colonic fermentation assay in both extracts, the positive control, and the negative control as a means of proteolytic activity and metabolic functionality of gut microbiota. Thus, a high level of ammonium in the colon has been associated with an imbalanced state of colon microbial communities, leading to a pro-inflammatory situation with negative effects on host health.^66^ Notwithstanding, considering *in vitro* fermentation conditions, a progressive increase in ammonium ion production was expected.^67^

In this study, a positive control (inulin) led to the lowest increase in NH_4_^+^ production at 24 and 48 h (Table 4). However, no clear differences were found between negative control and plant extracts.

**Table 4:** Ammonium Ion Concentration (mg/L) during *In Vitro* Colonic Fermentation of Yarrow Extract (YE), Marjoram Extract (ME), and Positive and Negative Controls

	YE	ME	Positive control	Negative control
**0 h**	64.66 ± 9.37[Table-fn tbl4fn1] ^a^_A_	70.45 ± 7.33^a^_A_	65.79 ± 3.42^a^_A_	67.75 ± 4.90^a^_A_
**24 h**	326.24 ± 5.56^b^_B_	355.09 ± 25.21^b^_C_	210.32 ± 5.75^b^_A_	379.24 ± 7.23^b^_C_
**48 h**	439.32 ± 5.12^c^_C_	436.03 ± 12.34^c^_C_	214.12 ± 7.12^b^_A_	391.03 ± 4.51^b^_B_

I Mean ± standard deviation. Uppercase letters denote statistical differences (*p*-value < 0.05) between samples at the same time point. Lowercase letters mean statistical differences (*p*-value < 0.05) between time points within each sample.

A lower content of ammonium was hardly shown by YE at 24 h compared to ME or the negative control, whereas a slightly higher level was noticed for both extracts compared to the negative control at 48 h. In this context, a high level of proteolytic activity of microbiota could be related to a limited availability of fermentable carbohydrates in the fermentative medium.^68^ However, it is not possible to determine the specific cause of this increase, since there are a limited number of studies evaluating the effect of an acute intake of pure phenolic compounds or extracts on ammonium production. Nevertheless, several studies have reported a decrease of this compound following a chronic consumption of PCs from tea, cranberry, or grapes.^21,39,69^

Moreover, SCFA (acetic, propionic, and butyric acids) content (Figure 2) was measured in both extracts, the positive control, and the negative control as an estimation of microbial fermentative activity and metabolic functionality.

**Figure 2:**
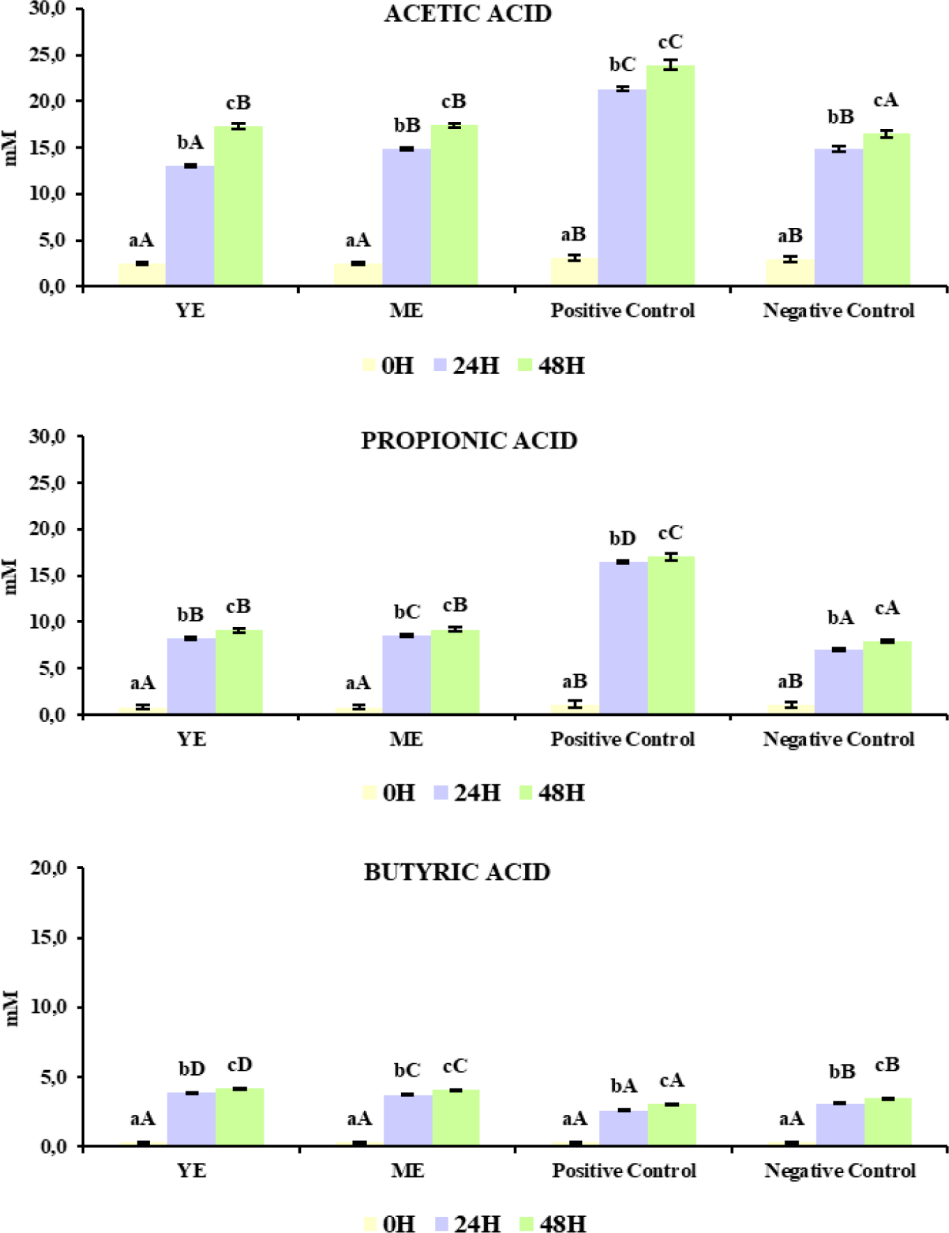
Short-chain fatty acids (acetic, propionic, and butyric acid) content in the fermentation samples of yarrow and marjoram extracts and positive and negative controls. Data include the mean (mM) ± standard deviation. Uppercase letters denote statistical differences (*p*-value < 0.05) between yarrow, marjoram, positive control, and negative control within the time point and bacterial group. Lowercase means statistical differences (*p*-value < 0.05) between time points within samples and bacterial groups.

SCFAs play a key role in maintaining intestinal homeostasis and gut health.^9^ Significant increases (*p*-value < 0.05) in this SCFA production at 24 and 48 h were observed in all fermentation samples compared to 0 h, with acetic acid production standing out, especially for the positive control (6% regarding time 0 h). Regarding the plant extracts, colonic fermentation at 48 h resulted in a production of acetic acid higher than in the negative control. The same trend was observed for propionic acid since the extracts generated an increase in its production (≈9%), lower than the positive control (≈ 14%), but higher than the negative control (≈ 6%). Interestingly, both PC extracts led to the production of butyric acid at 24 and 48 h (12% higher at 48 h), being significantly higher (*p*-value < 0.05) than both positive (≈ 7.5%) and negative (≈ 9%) controls. This was of great interest as butyric acid is the main energy source for colonocytes and is related to different health effects including antidiarrheal, antioxidant, anticancer, and anti-inflammatory properties.^70^ These results agree with Perez-Burillo et al.^71^ who also observed a significant increase in butyric acid production throughout the colonic fermentation assays of other phenolic sources such as green tea, olive leaf, rosemary, eucalyptus, etc. A possible explanation for this increase in butyric acid production may lie in the stimulation of butyric-acid-producing bacteria by phenolic compounds. The greater producer of butyric is the phylum Firmicutes, although some bacteria from the phyla Actinobacteriota, Bacteroidota, and Proteobacteria would also be producers of butyrate.^72^

### 3.4 Effect of Yarrow and Marjoram Extracts on Colonic Microbiota

The impact of digested YE and ME on gut microbiota during colonic fermentation was evaluated by plate counts as a first approach (Table S6). From a microbiological point of view, differences in values were considered significant when they were statistically significant and higher than Δlog (CFU/mL) ≥ 1 due to plate counting limitations.^27,39^

A significant increment in the total aerobes and anaerobes was recorded for all samples at 24 h. These microbial populations remained stable at 48 h, except for the YE where a slight decrease was observed. However, no changes in the plate counts of *Enterobacteriaceae*, *Enterococcus* spp., *Clostridium* spp., or *Bifidobacterium* spp. were found in any sample during the assay. In addition, there was a significant decrease in *Staphylococcus* spp., lactic acid bacteria, and *Lactobacillus* spp. in all samples at 24 h, except for the positive control, where no significant variation in the *Lactobacillus* spp. population was obtained during the assay. Moreover, a less pronounced decrease in the number of lactic acid bacteria was also shown for positive control samples.

Complementarily, 16S rRNA gene sequence analysis and subsequent calculation of microbial diversity indexes were developed on colony fermentation samples. Figure 3 shows the alpha-diversity in terms of observed ASVs (richness), Shannon (equity), and Simpson (dominance) indexes. No statistically significant differences were found between different treatments at 0, 24, and 48 h, probably due to the nature of samples and replicates, except for Simpson indexes, which revealed statistically significant differences between the positive control and yarrow at 24 h and positive and negative controls and marjoram at 48 h (*p*-value < 0.05). However, some interesting trends were observed. The initial values (0 h) showed the major diversity in terms of richness. It is important to emphasize the loss of diversity in YE samples at 24 h (*p*-value <0.1), although it seems to recover toward the end of the experiment. On the other hand, the diversity of ME samples was less at 48 h (*p*-value < 0.05).

**Figure 3:**
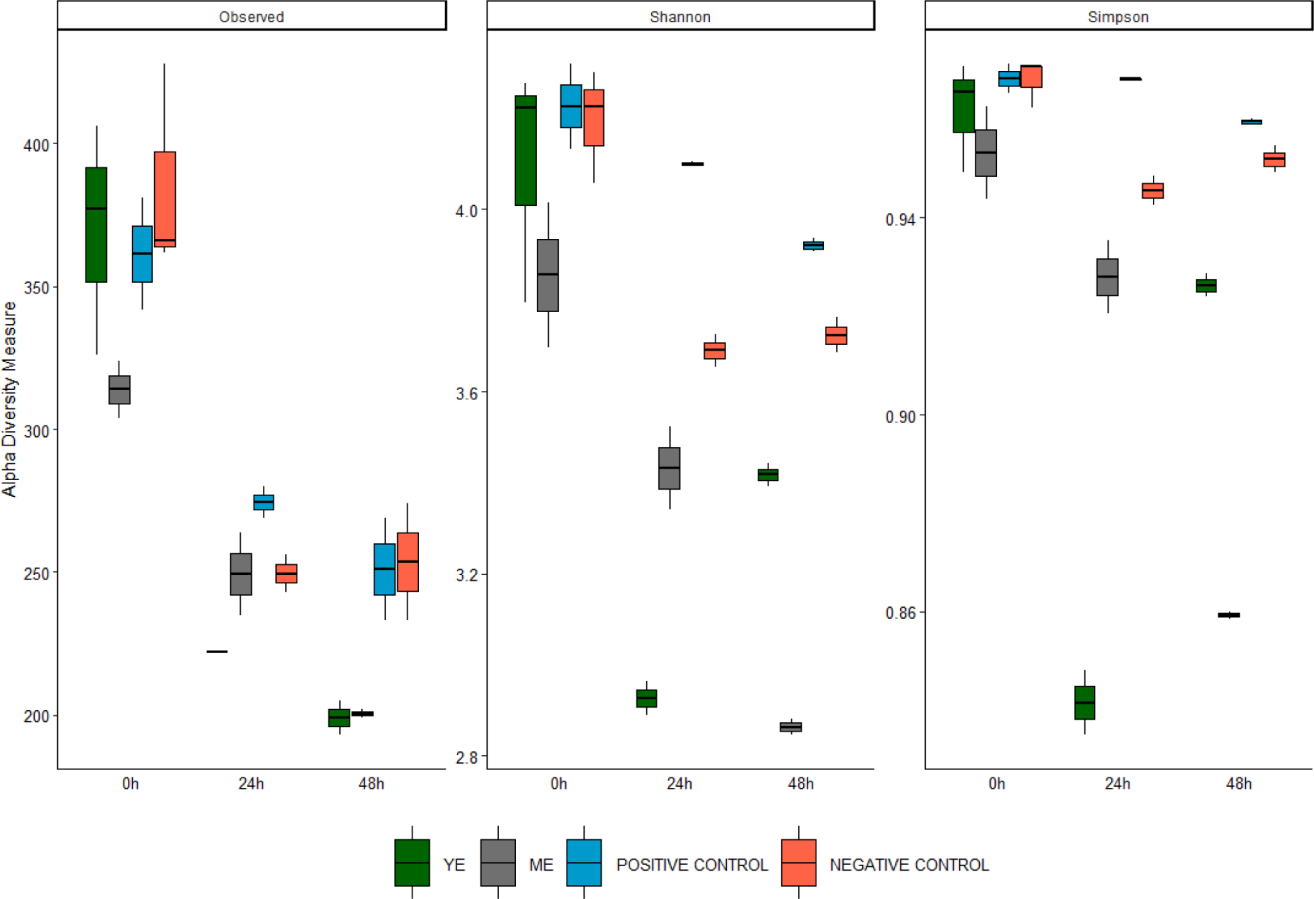
Alpha-diversity in terms of observed species and Shannon and Simpson indexes of the microbiota present in the fermentation samples of yarrow extract (YE) and marjoram extract (ME) and positive and negative controls at different times (0 h, 24 h, and 48 h). Center lines show the medians, box limits indicate the 25th and 75th percentiles, and whiskers extend 1.5 times the interquartile range from the 25th and 75th percentiles.

In line with the alpha-diversity results, beta-diversity analysis (Figure 4) showed an alignment or clustering of all fermentation samples at the initial time. However, at 24 and 48 h, both YE and ME fermentation samples clearly differed from both controls suggesting that digested extracts produced changes in the microbiota composition.

**Figure 4:**
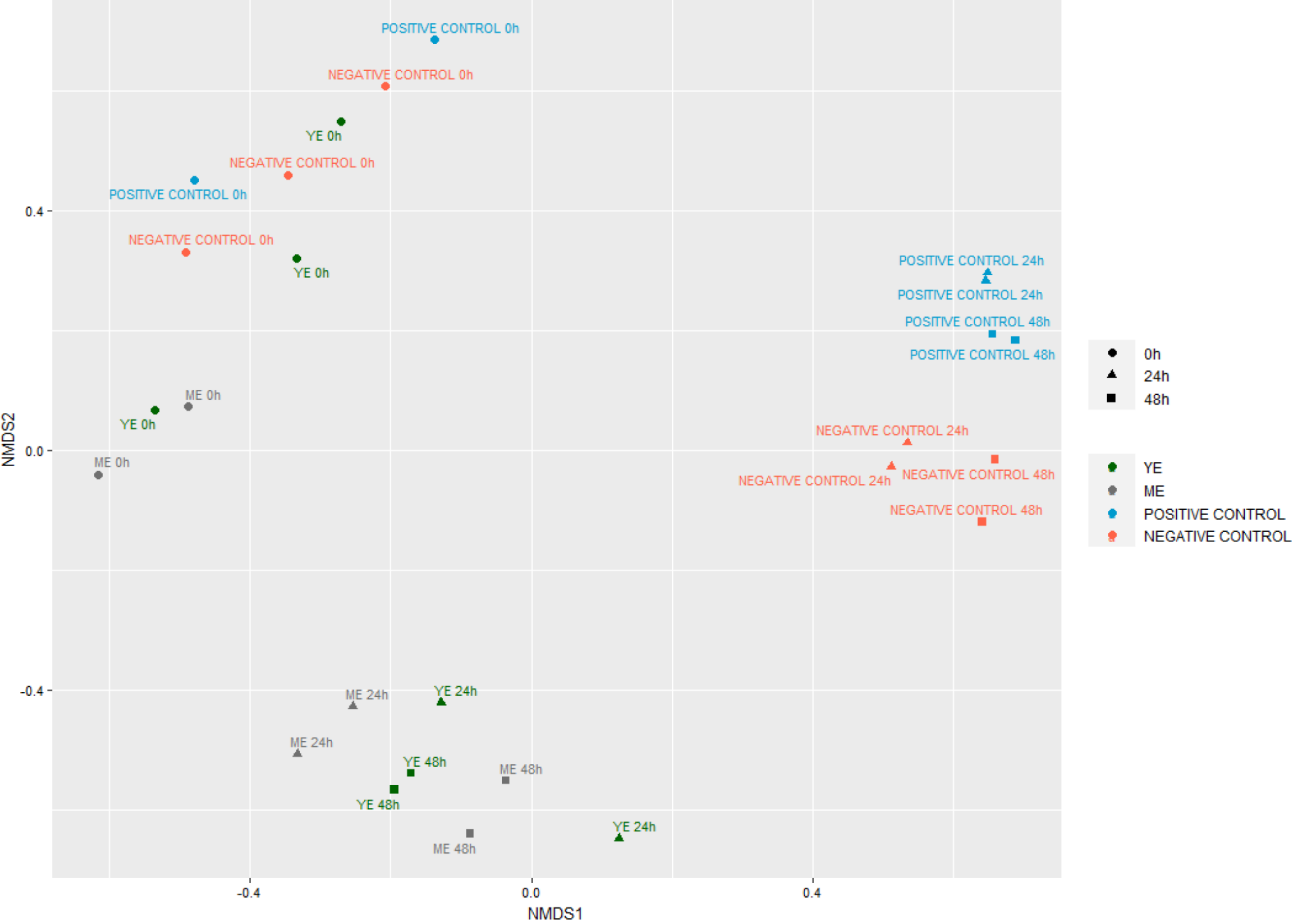
Nondimensional scaling (NMDS) representation of the microbial beta-diversity from the colonic fermentation samples of yarrow extract (YE) and marjoram extract (ME) and positive and negative controls at 0 h, 24 h, and 48 h.

Taxonomic analysis at the phylum and genus level was conducted in terms of relative abundance for those taxa with a mean relative abundance greater than 0.5% (Figure 5A,B). Statistical analysis revealed differences between assays for some taxa (Table S7). At the beginning of all fermentation assays, the predominant phyla were Firmicutes followed by Actinobacteriota. The minor phyla were Proteobacteria, Fusobacteria, Bacteroidota, Desulfobacteria, and Verrucomicrobiota. These have been described as the dominant gut microbiota phyla, of which Firmicutes and Bacteroidota account for more than 90%.^1^ The positive control, inulin, was employed as a well-known prebiotic compound. Its beneficial impact was observed through the significant increase in Actinobacteriota and Bacteroidota members (Table S7) and the reduction of Firmicutes phylum along the study (*p*-value < 0.1). These results are consistent with previous studies that have linked inulin to beneficial modulation of the microbiota.^73^ In the case of digested YE and ME, an increase in the relative abundance of Actinobacteriota was also observed at 48 h compared to time 0 h (*p*-value < 0.05).

**Figure 5:**
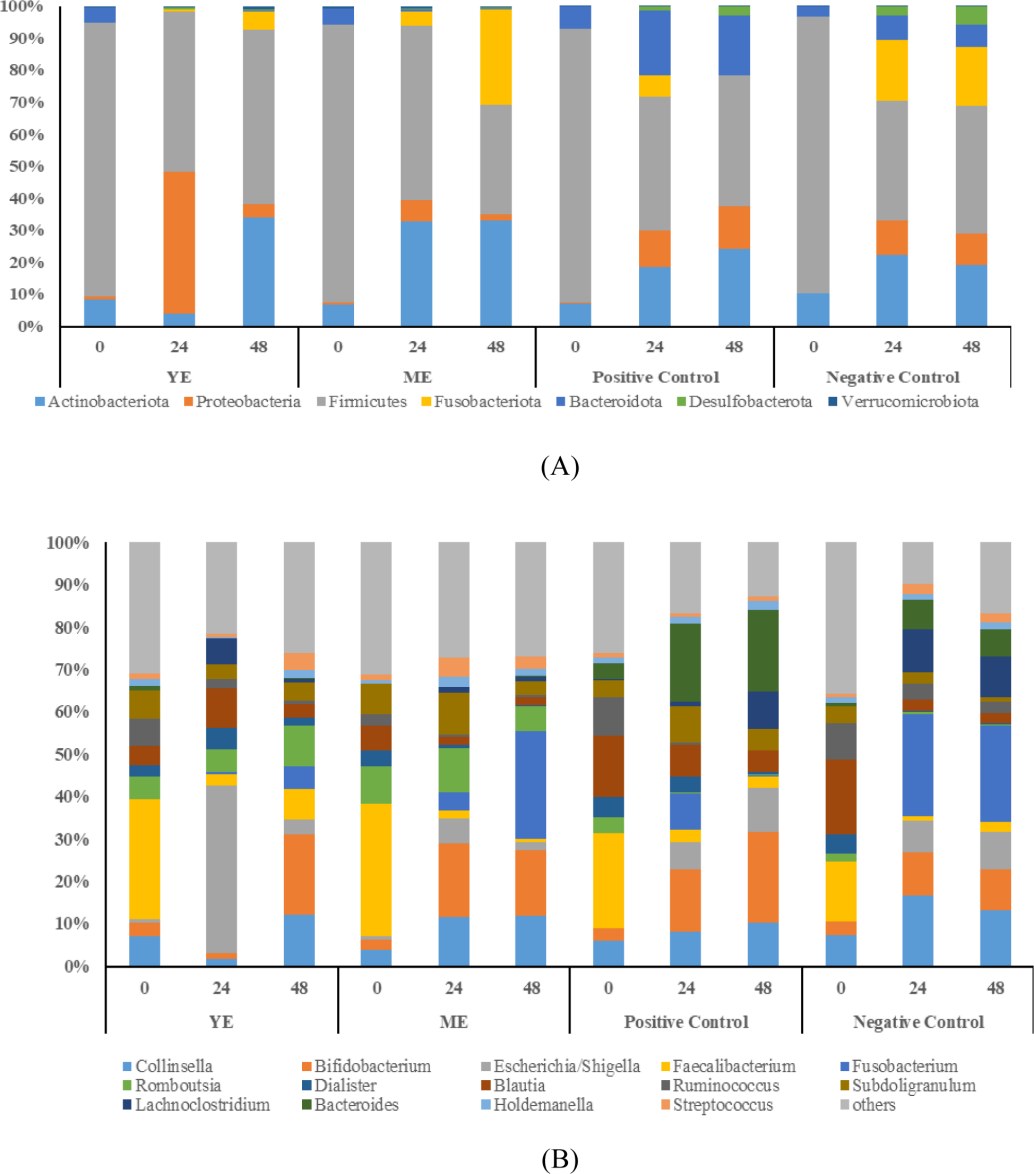
Relative abundance in phyla (A) and genus (B) of the microbiota present in the fermentation samples of yarrow extract (YE), marjoram extract (ME), and positive and negative controls at 0 h, 24 h, and 48 h.

More in detail, for ME, the relative abundance increased from 7% to 33% at 24 h and remained stable until 48 h; meanwhile, for YE, the increase in the relative abundance was observed at 48 h (9% to 34%). Regarding Proteobacteria, which have been associated with common pathogens,^1^ all samples assessed significantly promoted their growth at 24 h, with a reduction at 48 h although not to their basal levels. The lowest increase was observed for ME which increased from 0.7% to 2% at the end of the assay. In contrast, the results observed for the YE were remarkable. In this case, the Proteobacteria phylum members increased from a relative abundance of 0.4% to 44% at 24 h and finally to 4% at 48 h. This increase was mainly due to the statistically significant increase observed for the *Escherichia*/*Shigella* genus members, showing the same trend in growth evolution (Table S7). *Escherichia*/*Shigella* members are major drivers in *in vitro* fermentation processes, showing, in general, an increase during the first 24 h in control conditions due to their advances in growth mechanisms and growth benefits.^27,74^ Furthermore, some authors have pointed out that Gram-negative families (as *Escherichia*/*Shigella*) could be less sensitive to the antimicrobial effect of some PC sources as plant foods (grape and apples) or herbals (rosemary and oregano).^21,71^ This increase may even be associated with the presence of certain PCs, such as DCQAs.^75^

At the beginning of the assay, the bacterial genera with the highest relative abundance were *Collinsella*, *Bifidobacterium*, *Fusobacterium*, *Romboutsia*, *Dialister*, *Blautia*, *Ruminococcus*, *Agathobacter*, and *Subdoligranulum*. *Faecalibacterium*, *Ruminococcus*, and *Agathobacter* (all belonging to the Firmicutes phylum) decreased during the fermentation of all samples, although these trends did not show statistical significance (Table S7). *Fusobacterium* levels significantly increased with all samples, although a major stimulating effect was observed for ME at 48 h. The analysis of bacteria species revealed that this increment was mainly due to the significant increase in *Fusobacterium ulcerans* levels, a minority and less known species of this genus. In contrast, it is noteworthy the positive effect of the extracts on the following taxa. First, and in line with Actinobacteriota phylum results, the PC extracts of YE and ME allowed the meaningful increase in the *Bifidobacterium* genus members, the well-known probiotic group, even to a greater extent than the inulin positive control. The growth-stimulating effect for both plants was 8 times higher compared to the beginning of the assay although in the case of ME, it was at 24 h and for YE at 48 h. In line with our results, phenolic acids such as caffeic acid, DCQAs, or rosmarinic acid have shown their role in the stimulation *in vivo* of *Bifidobacterium*.^13,14,75^

On the other hand, *Collinsella* members, also belonging to the Actinobacteriota phylum, tended to enhance their relative abundance with all samples, especially with marjoram ME at 24 h and YE at 48 h. This bacterial genus was also stimulated by other plant extracts rich in PCs (olive, rosemary, oregano, and eucalyptus).^71^ An example of a genus belonging to the phylum Bacteroidota was *Blautia*. This genus, associated with the metabolism of PCs and possible beneficial properties in intestinal disorders,^76^ showed a 5-fold increase in the relative abundance after 24 h of YE. These results agree with the increasing production of butyrate obtained for both extracts, as these phyla are related to modulation of the microbiota by increased growth of butyrate-producing species,^77^ or to butyrate production and related health benefits.^72^

Positive effects on host health have been associated with the genera belonging to *Firmicutes*, *Dialister*, and *Romboutsia*. The *Dialister* genus, described as a propionate producer, was stimulated by 3% at 24 h in YE samples (*p*-value < 0.1). The relative abundance of *Romboutsia*, linked to hosts healthy status,^78^ significantly increased by 3% at 24 h with ME and 6% at 48 h with YE. A subsequent analysis of microbiota at the species level also revealed the growth of bacteria with positive effects on health. The most promising effect was observed for *Akkermansia muciniphila*, which is related to the maintenance of the intestinal barrier.^79^ *A. muciniphila* presented a meaningful increase during the colonic fermentation of both extracts, especially with YE at 48 h (Table S7). The increase in *A. muciniphila* agrees with previous *in vitro* fermentation studies performed with PC sources such as red wine, grape juice, and black tea.^38,68^ Moreover, this increment could be attributed to the presence of caffeic acid, CQAs, or rosmarinic acid as reported by other studies.^14,80^

In conclusion, this paper reports that the digestion process caused a significant decrease in PC bioaccessibility, although it was affected by the structure of the compound (e.g., 56% and 9% for orientin and sterubin, respectively) and plant extract (e.g., 20% vs. 43% for yarrow and marjoram, respectively). The subsequent colonic fermentation of the nonbioaccessible PCs drastically reduced their content, yielding phloroglucinol, 3,4-dimethoxyphenylacetic acid, 3-(4-hydroxyphenyl)-propionic acid, and 4-hydroxybenzoic acid as the main colonic metabolites by microbial bioconversion of CQAs, rosmarinic acid, and flavones, such as apigenin, luteolin, their derivatives, or the hydroxymethoxyflavones.

Parallel to the phenolic metabolism, incubations in the presence of extracts led to changes in the gut bacteriome. These changes were more apparent at 24 h for marjoram extract and 48 h for yarrow. The main stimulatory effects were those observed for *Bifidobacterium*, *Collinsella*, *Romboutsia*, and *Akkermansia muciniphila* members for both plant extracts and *Blautia* and *Dialister* for yarrow extract, and all of them were related to positive health effects. These changes could be related to an increase in certain SCFAs, mainly butyric acid, but also to the microbial conversion of rosmarinic acid or CQAs without ruling out the involvement of the various flavones present in the extracts. In relation to the experimental approach used, this study reinforces the use of static fermentation as a first tool to study the impact of the prebiotic potential of foods and food ingredients on the gut microbiota, including novel features related to the microbial catabolism of (poly)­phenols.^20,81,82^

In summary, this research shows the behavior of less studied phenolic compounds, such as luteolin, apigenin, and other hydroxymethoxyflavones, throughout gastrointestinal digestion and colonic fermentation. Moreover, it highlights the prebiotic-like effect of edible plants not studied such as marjoram and yarrow, especially the latter. Therefore, the consumption of these extracts, either in the form of dietary supplements, nutraceuticals, or their use as functional ingredients in food formulation, could exert a beneficial effect on the human gut microbiota in foods as functional ingredients.

## Supplementary Material



## Notes

Table S1: phenolics identified by HPLC-PAD-ESI-QTOF-MS/MS (negative ionization mode) in the UAE yarrow extract; Table S2: phenolics identified by HPLC-PAD-ESI-QTOF-MS/MS (positive ionization mode) in the UAE yarrow extract; Table S3: phenolic compounds identified by HPLC-PAD-ESI-QTOF-MS/MS (negative ionization mode) in the UAE marjoram extract; Table S4: content of phenolic compounds of yarrow extract (YE), pH-simulated gastrointestinal digestion phases, and residues after simulated gastrointestinal digestion without enzymes and bile salts (mg/g dry extract); Table S5: phenolic compound content of marjoram extract (ME), pH-simulated gastrointestinal digestion phases, and residues after simulated gastrointestinal digestion without enzymes and bile salts (mg/g dry extract); Table S6: evolution of microbial growth on the selected bacterial groups population during *in vitro* colonic fermentation of yarrow extract (YE), marjoram extract (ME), and positive and negative controls; Table S7: statistical analysis to assess taxa differences among taxa between marjoram extract (ME), yarrow extract (YE), and positive and negative control and times at phylum, genus, and species level (PDF)
