# Supplementary material for: Modulation of Gut Microbiota Composition and Microbial Phenolic Catabolism of Phenolic Compounds from Achillea millefolium L. and Origanum majorana L
Source: J Agric Food Chem. 2024 Dec 19;73(1):478–94. doi: 10.1021/acs.jafc.4c07910 (PMC12506861; doi:10.1021/acs.jafc.4c07910)
Supplement: Supplementary file 1 [file jf4c07910_si_001.pdf]

Supporting Information

Modulation of gut microbiota composition and microbial phenolic catabolism of phenolic compounds from *Achillea millefolium* L. and *Origanum majorana* L.

Irene Fernandez-Jalao[a,b], María de las Nieves Siles-Sánchez[a,b], Susana Santoyo[a,b†], Alba Tamargo[c], Edgard Relaño de la Guía[c], Natalia Molinero[c], Victoria Moreno-Arribas[c], Laura Jaime[a,b†*].

[a]Departmental section of Food Science, Faculty of Science, Universidad Autónoma de Madrid, 28049 Madrid, Spain.

[b] Department of Production and Characterization of Novel Food, Food Science Research Institute (CIAL), CEI CSIC-UAM, 28049 Madrid, Spain.

[c]Department of Food Biotechnology and Microbiology, Food Science Research Institute (CIAL), CEI CSIC-UAM, 28049 Madrid, Spain.

† Authors have equally contributed to this study.

*Corresponding author:

Email: laura.jaime@uam.es (Laura Jaime)

Table S1. Phenolics identified by HPLC-PAD-ESI-QTOF-MS/MS (negative ionization mode) in the UAE yarrow extract.

Rt (min)	Compound	Theoretical Mass *(m/z)*	Experimental Mass *(m/z)*	MS/MS product ions *(m/z)*
**Hydroxyccinamic acids and derivatives**				
11.32	Neochlorogenic acid	353.08726	353.08847	191 (100), 179 (72)
13.44	Chlorogenic acid	353.08726	353.08908	191 (100)
13.63	Cryptochlorogenic acid	353.08726	353.08908	135 (100), 179 (30)
16.50	Caffeic acid	179.03444	179.03482	135 (100)
24.12	3,4- Dicaffeoylquinic acid	515.11896	515.12103	515 (43), 353 (100), 173 (79), 179 (47)
25.36	1,5- Dicaffeoylquinic acid	515.11896	515.12146	191 (100), 192 (10)
25.58	3,5- Dicaffeoylquinic acid	515.11896	515.12158	191 (100), 179 (53), 135 (34)
26.87	4,5- Dicaffeoylquinic acid	515.11896	515.12115	353 (100), 354 (23), 173 (54), 179 (38)
29.05	1,4-Dicaffeoylquinic acid	515.11896	515.12054	353 (100)
**Flavone derivatives**				
14.36	Vicenin II	593.15065	593.15466	593 (100), 473 (9)
15.97	Schaftoside isomer	563.14009	563.14423	563 (100), 473 (8)
16.76	Schaftoside	563.14009	563.14423	563 (100), 473 (8)
17.43	Homoorientin	447.09274	447.09552	447 (44), 429 (20), 357 (100), 327 (91), 285 (10)
17.87	Apigenin- hexoside- pentoside	563.14009	563.14374	563 (100), 473 (8)
18.13	Trihydroxy-methoxy flavone hexoside	461.10839	461.11200	313 (100)
18.16	Luteolin-hexoside	609.14557	609.14935	609 (100), 285 (19)
18.50	6-hydroxyluteolin 7-*O*-glucoside	463.08766	463.09073	463 (100), 301 (95), 300 (36)
21.08	Vitexin	431.09783	431.10074	431 (359, 311 (100), 341 (60)
22.07	Methylapigenin-hexoside	445.11348	445.11649	445 (100), 297 (9)
22.27	Luteolin 7-*O*-glucoside	447.09274	447.09613	285 (90); 284 (100)
23.29	Hydroxymethoxyflavone-hexoside	475.12404	475.12720	475 (100), 460 (26), 327 (8)
26.22	Apigenin 7-*O*-glucoside	431.09783	431.09967	269 (100)
27.35	Trihydroxyflavone-hexuronide	445.07709	445.07993	269 (100)
32.30	Luteolin	285.03992	285.04169	285 (11), 151 (100), 133 (96)
35.71	Apigenin	269.04500	269.04633	269 (100), 151 (16), 149 (12)

Table S1- Continued

36.47	Diosmetin	299.05557	299.05637	299 (39), 284 (100)
40.85	Dihydroxydimethoxyflavone	313.07122	313.07309	311 (23), 283 (100), 298 (57)
42.01	Dihydroxytrimethoxyflavone	343.08178	343.08383	343 (14), 328 (100), 313 (82)
**Flavanols**				
19.68	Quercetin-hexoside	463.08766	463.09103	463 (100), 302 (25), 301 (76)
20.52	Rutin	609.14557	609.14856	609 (15), 300 (100), 301 (12)
32.58	Quercetin	301.03483	301.03583	301 (33), 151 (100)
**Flavanones**				
32.00	Eriodyctiol	287.05557	287.05707	135 (100), 134 (16)
35.66	Naringenin	271.06065	271.04654	151 (74), 119 (100), 107 (22)
**Flavanonols**				
24.48	Taxifolin	303.05048	303.05191	285 (29), 153 (9), 125 (100)

Table S2. Phenolics identified by HPLC-PAD-ESI-QTOF-MS/MS (positive ionization mode) in the UAE yarrow extract.

**Rt (min)**	**Compound**	**Theoretical Mass *(m/z)***	**Experimental Mass *(m/z)***	**MS/MS product ions *(m/z)***
Flavone derivatives				
45.65	Hydroxytetramethoxyflavone	359.11308	359.11380	344 (100), 329 (79)
46.43	Hydroxytrimethoxyflavone	329.10252	329.10270	314 (57), 299 (100)

Table S3. Phenolic compounds identified by HPLC-PAD-ESI-QTOF-MS/MS (negative ionization mode) in the UAE marjoram extract.

**Rt (min)**	**Compound**	**Theoretical Mass *(m/z)***	**Experimental Mass *(m/z)***	**MS/MS product ions *(m/z)***
**Hydroquinone derivatives**				
8.60	Arbutin	271.08178	271.08487	108 (100), 71 (11)
**Hydroxyccinamic acids and derivatives**				
16.50	Caffeic acid	179.03444	179.03482	135 (100)
17.18	Caffeoylarbutin isomer I	433.11348	433.11627	433 (88), 179 (18), 161 (100)
27.63	Rosmarinic acid	359.07670	359.07886	161 (100), 197 (34), 179 (19)
29.03	Lithospermic acid isomer	537.10331	537.10645	135 (100), 161 (47), 179 (25), 197 (20)
29.28	Salvianolic acid isomer	717.14557	717.14856	321 (100), 339 (39)
**Flavone derivatives**				
14.36	Vicenin II	593.15065	593.15466	593 (100), 473 (9)
17.43	Homoorientin	447.09274	447.09552	447 (44), 429 (20), 357 (100), 327 (91), 285 (10)
18.46	Orientin	447.09274	447.090528	327 (100), 357 (27), 447 (21)
22.27	Luteolin 7-*O*-glucoside	447.09274	447.09613	285 (90); 284 (100)
22.74	Luteolin 7-*O*-glucuronide	461.07201	461.07465	461 (11), 285 (100)
26.82	Apigenin 7-*O*-glucuronide	445.0779	445.0686	445 (20), 269 (100)
32.30	Luteolin	285.03992	285.04169	285 (11), 151 (100), 133 (96)
33.15	Trihydroxymethoxyflavone	299.05557	299.05707	299 (29), 284 (100)
33.96	Trihydroxydimethoxyflavone I	329.06613	329.06778	329 (19), 314 (100), 299 (37)
35.27	Trihydroxy-dimethoxy flavone II	329.06613	329.06784	329 (18), 314 (51), 299 (100)
35.71	Apigenin	269.04500	269.04633	269 (100), 151 (16), 149 (12)
36.12	Trihydroxy-trimethoxy flavone I	359.07670	359.07864	344 (100), 329 (99)
**Flavanone derivatives**				
32.00	Eriodyctiol	287.05557	287.05707	135 (100), 134 (16)
35.66	Naringenin	271.06065	271.04654	151 (74), 119 (100), 107 (22)
39.46	Sterubin	301.07122	301.07300	135 (100), 165 (36)
**Flavanonol derivatives**				
24.48	Taxifolin	303.05048	303.05191	285 (29), 153 (9), 125 (100)

Table S4: Content of phenolic compounds of yarrow extract (YE), pH simulated gastrointestinal digestion phases, and residues after simulated gastrointestinal digestion without enzymes and bile salts (mg/g dry extract).

	Gastrointestinal digestion samples				
	**YE**	**Gastric phase**	**Intestinal phase**	**Residue after digestion**	**Loss (%)**
**<u>Phenolic acids</u>**					
**Hydroxycinnamic acids**					
Neochlorogenic acid	0.26 ± 0.01*[c]	0.19 ± 0.01[a]	0.23 ± 0.00[b]	n.d.	12
Chlorogenic acid	2.68 ± 0.02[d]	2.27 ± 0.03[b]	2.59 ± 0.01[c]	0.03 ± 0.00[a]	2
Cryptochlorogenic acid	0.06 ± 0.00[a]	0.06 ± 0.00[a]	0.18 ± 0.01[b]	n.d.	-
Caffeic acid	0.21 ± 0.01[bc]	0.14 ± 0.00[a]	0.19 ± 0.03[b]	n.d.	10
3,4-dicaffeoylquinic acid	0.94 ± 0.01[c]	0.57 ± 0.02[b]	0.96 ± 0.09[c]	0.08 ± 0.01[a]	-
1,5-dicaffeoylquinic acid	0.81 ± 0.01[c]	0.45 ± 0.05[b]	0.51 ± 0.04[b]	0.17 ± 0.01[a]	16
3,5-dicaffeoylquinic acid	10.97 ± 0.09[c]	7.10 ± 0.60[b]	7.26 ± 0.31[b]	1.49 ± 0.10[a]	20
4,5-dicaffeoylquinic acid	1.91 ± 0.04[d]	1.43 ± 0.04[b]	1.72 ± 0.18[cd]	0.16 ± 0.02[a]	2
**<u>Flavonoids</u>**					
**Flavones**					
Vicenin II	5.83 ± 0.06[d]	3.97 ± 0.09[b]	4.38 ± 0.11[c]	0.25 ± 0.00[a]	21
Schaftoside acid isomer I	1.19 ± 0.08[c]	0.89 ± 0.04[b]	0.92 ± 0.03[b]	0.12 ± 0.00[a]	13
Schaftoside acid isomer II	2.94 ± 0.05[c]	2.10 ± 0.12[b]	2.21 ± 0.05[b]	0.17 ± 0.00[a]	19
Schaftoside acid	5.12 ± 0.09[c]	3.83 ± 0.20[b]	4.12 ± 0.10[b]	0.13 ± 0.01[a]	17
Homoorientine	0.47 ± 0.01[c]	0.34 ± 0.02[b]	0.37 ± 0.03[b]	0.04 ± 0.00[a]	13
Apigenin-hexoside-pentoside	2.92 ± 0.03[c]	2.11 ± 0.04[b]	2.19 ± 0.06[b]	0.18 ± 0.01[a]	19
Luteolin-hexoside	3.37 ± 0.04[d]	2.17 ± 0.06[b]	2.35 ± 0.07[c]	0.19 ± 0.02[a]	25
Hydroxyluteolin-hexoside	2.64 ± 0.03[d]	1.39 ± 0.07[b]	1.45 ± 0.05[c]	0.23 ± 0.01[a]	36
Vitexin	1.02 ± 0.01[d]	0.70 ± 0.01[b]	0.78 ± 0.03[bc]	0.11 ± 0.00[a]	13
Methylapigenin-hexoside	6.11 ± 0.06[c]	3.96 ± 0.19[b]	3.98 ± 0.15[b]	0.49 ± 0.07[a]	27
Luteolin-7-*O*-glucoside	5.48 ± 0.05[d]	3.28 ± 0.08[b]	4.11 ± 0.07[c]	0.34 ± 0.04[a]	19
Hydroxydimethoxyflavone-hexoside	0.76 ± 0.01[c]	0.55 ± 0.01[b]	0.52 ± 0.02[b]	0.16 ± 0.00[a]	11
Apigenin-7-*O*-glucoside	1.14 ± 0.03[c]	0.63 ± 0.03[b]	0.69 ± 0.05[b]	0.23 ± 0.04[a]	19
Trihydroxyflavone-hexuronide	0.93 ± 0.05[c]	0.48 ± 0.03[b]	0.53 ± 0.03[b]	0.27 ± 0.01[a]	14

Luteolin	1.28 ± 0.01^{c}	0.73 ± 0.04^{b}	0.77 ± 0.06^{b}	0.38 ± 0.03^{a}	10
Apigenin	0.38 ± 0.02^{c}	0.22 ± 0.01^{b}	0.24 ± 0.03^{b}	0.13 ± 0.01^{a}	3
Diosmetin	0.45 ± 0.00^{d}	0.21 ± 0.02^{b}	0.27 ± 0.02^{bc}	0.14 ± 0.01^{a}	9
Dihydroxydimethoxyflavone	2.03 ± 0.03^{c}	0.78 ± 0.04^{b}	0.84 ± 0.03^{b}	1.03 ± 0.05^{a}	8
Dihydroxytrimethoxyflavone	2.76 ± 0.11^{d}	1.23 ± 0.04^{b}	1.32 ± 0.05^{bc}	1.12 ± 0.06^{a}	12
Hydroxytetramethoxyflavone	4.60 ± 0.05^{c}	2.26 ± 0.12^{b}	2.44 ± 0.09^{b}	1.54 ± 0.09^{a}	13
Hydroxytrimethoxyflavone	1.68 ± 0.02^{c}	0.65 ± 0.01^{a}	0.71 ± 0.05^{ab}	0.73 ± 0.03^{ab}	14
**Flavonols**					
Quercetin-hexoside	0.99 ± 0.10^{d}	0.52 ± 0.02^{b}	0.65 ± 0.02^{c}	0.17 ± 0.00^{a}	17
Rutin	1.51 ± 0.02^{d}	1.15 ± 0.03^{b}	1.23 ± 0.03^{bc}	0.09 ± 0.00^{a}	13
Quercetin	0.26 ± 0.02^{cd}	0.16 ± 0.02^{b}	0.21 ± 0.01^{c}	0.06 ± 0.01^{a}	-
**Σ Phenolic compounds**	**73.70**	**46.52**	**50.92**	**10.23**	17

*Data are expressed as mean ± standard deviation (n=3). Superscript letters mean statistically significant differences (*p*-value < 0.05) between extract and gastrointestinal digestion phases (gastric phase, intestinal phase) and residue after gastrointestinal digestion. n.d. (not detected).

Table S5: Phenolic compounds content of marjoram extract (ME), pH simulated gastrointestinal digestion phases and residues after simulated gastrointestinal digestion without enzymes and bile salts (mg/g dry extract).

	Gastrointestinal digestion samples				
	**ME**	**Gastric phase**	**Intestinal phase**	**Residue after digestion**	**Loss (%)**
**Simple phenols**					
Arbutin	67.98 ± 1.13*^{cd}	63.68 ± 2.93^{bc}	59.34 ± 1.45^{b}	1.43 ± 0.32^{a}	11
**Phenolic acids**					
**Hydroxycinnamic acids**					
Caffeic acid	0.43 ± 0.01^{b}	0.32 ± 0.01^{a}	0.42 ± 0.04^{b}	n.d.	2
Caffeoylarbutin isomer	2.26 ± 0.04^{c}	1.63 ± 0.06^{b}	1.61 ± 0.07^{b}	0.11 ± 0.01^{a}	24
Rosmarinic acid	38.12 ± 0.76^{d}	35.77 ± 1.29^{c}	28.37 ± 1.21^{b}	1.24 ± 0.13^{a}	22
Lithospermic acid isomer	7.21 ± 0.03^{d}	6.40 ± 0.41^{c}	3.71 ± 0.07^{b}	0.05 ± 0.01^{a}	48
Salvianolic acid isomer	1.87 ± 0.01^{c}	1.84 ± 0.05^{c}	1.53 ± 0.06^{b}	0.03 ± 0.00^{a}	17
**Flavonoids**					
**Flavones**					
Vicenin II	4.49 ± 0.03^{bc}	3.97 ± 0.09^{b}	4.03 ± 0.11^{b}	0.06 ± 0.01^{a}	9
Isorientin	0.40 ± 0.01^{a}	0.43 ± 0.04^{ab}	0.39 ± 0.02^{a}	n.d.	3
Orientin	0.62 ± 0.01^{b}	0.57 ± 0.01^{a}	0.55 ± 0.02^{a}	n.d.	11
Luteolin-7-*O*-glucoside	1.11 ± 0.02^{bc}	1.01 ± 0.07^{b}	0.98 ± 0.04^{b}	0.03 ± 0.00^{a}	9
Luteolin-7-*O*-glucuronide	3.90 ± 0.01^{c}	3.57 ± 0.08^{bc}	3.54 ± 0.10^{b}	0.04 ± 0.01^{a}	8
Apigenin-7-*O*-glucuronide	2.80 ± 0.05^{c}	2.69 ± 0.07^{b}	2.65 ± 0.04^{b}	0.05 ± 0.00^{a}	4
Luteolin	0.15 ± 0.00^{bc}	0.14 ± 0.02^{b}	0.14 ± 0.01^{b}	0.01 ± 0.00^{a}	-
Trihydroxymethoxyflavone	1.02 ± 0.02^{c}	1.04 ± 0.06^{c}	0.27 ± 0.01^{b}	0.10 ± 0.01^{a}	64
Trihydroxydimethoxyflavone I	2.67 ± 0.05^{d}	2.28 ± 0.12^{bc}	2.07 ± 0.14^{b}	0.18 ± 0.06^{a}	16
Trihydroxydimethoxyflavone II	0.75 ± 0.01^{d}	0.65 ± 0.01^{c}	0.23 ± 0.01^{b}	0.20 ± 0.01^{a}	43
Apigenin	0.04 ± 0.00^{b}	0.04 ± 0.00^{b}	0.04 ± 0.00^{b}	0.01 ± 0.00^{a}	-
Trihydroxytrimethoxyflavone	3.54 ± 0.08^{d}	3.12 ± 0.07^{c}	2.08 ± 0.06^{b}	0.13 ± 0.01^{a}	38
**Flavanones**					
Eriodyctiol	1.06 ± 0.02^{bc}	0.96 ± 0.11^{b}	0.92 ± 0.07^{b}	0.02 ± 0.00^{a}	11
Sterubin	3.07 ± 0.06^{c}	2.63 ± 0.18^{b}	2.50 ± 0.17^{ab}	0.37 ± 0.05^{a}	7

Table S5- Continued

**Flavanonols**					
Taxifolin	2.27 ± 0.03^{d}	2.12 ± 0.08^{c}	1.45 ± 0.02^{b}	0.06 ± 0.02^{a}	33
**Σ Phenolic compounds**	**146.24**	**135.23**	**117.16**	**4.15**	17

*Data are expressed as mean ± standard deviations (n=3). Lower case letters mean statistically significant differences (*p*-value < 0.05) between extract and gastrointestinal digestion phases (gastric phase, intestinal phase) and residue after gastrointestinal digestion. n.d. (not detected).

Table S6. Evolution of microbial growth on the selected bacterial groups population during *in vitro* colonic fermentation of yarrow extract (YE), marjoram extract (ME), positive and negative controls.

	Time (h)	**YE**	**ME**	**Positive Control**	**Negative Control**
**Total Aerobe**	0	5.12 ± 0.69*$^{a}_{A}$	5.82 ± 0.27$^{a}_{A}$	6.04 ± 0.04$^{a}_{A}$	6.03 ± 0.10$^{a}_{A}$
	24	7.68 ± 0.25$^{b}_{A}$	7.59 ± 0.06$^{b}_{A}$	7.21 ± 0.15$^{b}_{A}$	7.40 ± 0.06$^{b}_{A}$
	48	7.03 ± 0.14$^{b}_{A}$	7.29 ± 0.09$^{b}_{A}$	7.04 ± 0.05$^{b}_{A}$	7.31 ± 0.27$^{b}_{A}$
**Total Anaerobe**	0	5.39 ± 0.13$^{a}_{A}$	5.39 ± 0.05$^{a}_{A}$	5.51 ± 0.05$^{a}_{A}$	5.69 ± 0.07$^{a}_{A}$
	24	7.76 ± 0.01$^{c}_{A}$	7.59 ± 0.04$^{b}_{A}$	7.18 ± 0.05$^{b}_{A}$	7.49 ± 0.05$^{b}_{A}$
	48	6.88 ± 0.18$^{b}_{A}$	7.19 ± 0.07$^{b}_{A}$	6.99 ± 0.13$^{b}_{A}$	7.42 ± 0.23$^{b}_{A}$
Enterobacteriaceae	0	8.58 ± 0.06$^{a}_{A}$	8.61 ± 0.03$^{a}_{A}$	8.84 ± 0.19$^{b}_{A}$	8.71 ± 0.17$^{a}_{A}$
	24	8.25 ± 0.03$^{a}_{A}$	8.31 ± 0.11$^{a}_{A}$	7.99 ± 0.07$^{a}_{A}$	8.27 ± 0.07$^{a}_{A}$
	48	8.03 ± 0.13$^{a}_{A}$	8.06 ± 0.08$^{a}_{A}$	8.17 ± 0.03$^{ab}_{A}$	8.28 ± 0.20$^{a}_{A}$
***Enterococcus* spp.**	0	2.98 ± 0.22$^{a}_{A}$	3.15 ± 0.07$^{a}_{A}$	4.44 ± 0.08$^{a}_{B}$	4.35 ± 0.09$^{a}_{B}$
	24	2.96 ± 0.29$^{a}_{A}$	2.56 ± 0.74$^{a}_{A}$	4.34 ± 0.07$^{a}_{B}$	3.46 ± 0.56$^{a}_{AB}$
	48	3.15 ± 0.42$^{a}_{A}$	3.06 ± 0.45$^{a}_{A}$	5.43 ± 0.31$^{b}_{B}$	3.48 ± 0.56$^{a}_{A}$
***Clostridium* spp.**	0	7.93 ± 0.16$^{a}_{A}$	7.88 ± 0.14$^{a}_{A}$	7.27 ± 0.15$^{a}_{A}$	7.72 ± 0.14$^{a}_{A}$
	24	7.91 ± 0.18$^{a}_{A}$	7.75 ± 0.12$^{a}_{A}$	7.94 ± 0.18$^{a}_{A}$	8.13 ± 0.20$^{a}_{A}$
	48	7.40 ± 0.12$^{a}_{A}$	7.31 ± 0.04$^{a}_{A}$	7.83 ± 0.03$^{a}_{A}$	7.64 ± 0.17$^{a}_{A}$
***Staphylococcus* spp.**	0	3.72 ± 0.42$^{b}_{A}$	4.09 ± 0.21$^{b}_{AB}$	4.78 ± 0.10$^{b}_{B}$	4.71 ± 0.13$^{b}_{B}$
	24	2.52 ± 0.00$^{a}_{A}$	2.76 ± 0.24$^{a}_{A}$	2.91 ± 0.09$^{a}_{A}$	3.27 ± 0.39$^{a}_{A}$
	48	2.87 ± 0.44$^{ab}_{A}$	2.49 ± 0.86$^{a}_{A}$	2.62 ± 0.55$^{a}_{A}$	3.33 ± 0.43$^{a}_{A}$
**Lactic Bacteria**	0	7.20 ± 0.10$^{b}_{A}$	7.27 ± 0.09$^{b}_{A}$	7.40 ± 0.06$^{c}_{A}$	7.43 ± 0.02$^{b}_{A}$
	24	2.56 ± 0.74$^{a}_{A}$	2.58 ± 0.75$^{a}_{A}$	6.07 ± 0.08$^{b}_{B}$	3.24 ± 0.65$^{a}_{A}$
	48	3.04 ± 0.36$^{a}_{A}$	2.92 ± 0.70$^{a}_{A}$	4.53 ± 0.07$^{a}_{B}$	3.04 ± 1.06$^{a}_{A}$
***Lactobacillus* spp.**	0	5.34 ± 0.54$^{b}_{A}$	5.08 ± 0.09$_{A}$	5.04 ± 0.12$^{a}_{A}$	4.95 ± 0.15$^{b}_{A}$
	24	4.39 ± 0.04$^{a}_{A}$	0	5.74 ± 0.05$^{a}_{B}$	4.77 ± 0.02$^{b}_{AB}$
	48	0	0	5.42 ± 0.27$^{a}_{B}$	1.88 ± 0.32$^{a}_{A}$
***Bifidobacterium* spp.**	0	7.49 ± 0.09$^{a}_{A}$	7.50 ± 0.07$^{a}_{A}$	7.58 ± 0.07$^{a}_{A}$	7.60 ± 0.01$^{a}_{A}$
	24	7.55 ± 0.01$^{a}_{A}$	7.58 ± 0.07$^{a}_{A}$	7.58 ± 0.10$^{a}_{A}$	7.51 ± 0.13$^{a}_{A}$
	48	7.45 ± 0.15$^{a}_{A}$	7.27 ± 0.04$^{a}_{A}$	7.60 ± 0.02$^{a}_{A}$	7.38 ± 0.02$^{a}_{A}$

*Data are expressed as mean values of log (CFU/mL) ± standard deviations. Uppercase letters denote statistical differences (Δlog > 1) between samples at the same time point and bacterial group. Lower case letters mean statistical differences (Δlog > 1) between time points within each sample and bacterial group. CFU, colony forming units.

Table S7. Statistical analysis to assess taxa differences among taxa between marjoram extract (ME), yarrow extract (YE), positive and negative control and times at Phylum, Genus and Species level.

	Time 0 h				Time 24 h				Time 48 h			
**Taxa**	**Negative control**	**Positive Control**	**ME**	**YE**	**Negative control**	**Positive Control**	**ME**	**YE**	**Negative control**	**Positive Control**	**ME**	**YE**
**Phylum**												
Actinobacteriota	10.312b	7.264b	6.918b	8.582b	22.241ab	18.547b	32.670ab	3.990b	19.157ab	24.196ab	33.016a	34.084a
Proteobacteria	0.104c	0.187c	0.682c	0.822c	11.043b	11.483b	6.879bc	44.366a	9.896b	13.413ab	2.081c	4.023c
Firmicutes	86.260a	85.507a	86.398a	85.400a	37.054a	41.767a	54.190a	49.859a	39.809a	40.796a	34.072a	54.492a
Fusobacteriota	0.006c	0.016c	0.000c	0.018c	19.177b	6.667c	4.653c	0.665c	18.299b	0.138c	29.702a	5.674c
Bacteroidota	3.151c	6.842bc	5.177bc	4.767c	7.536abc	20.276a	0.553c	0.160c	7.114abc	18.643ab	0.382c	0.393c
Desulfobacterota	0.013d	0.028d	0.000d	0.035d	2.819ab	1.167abc	0.167cd	0.520bcd	5.591a	2.591ab	0.362cd	0.151d
Verrucomicrobiota	0.121c	0.137c	0.535abc	0.360c	0.086c	0.081c	0.811ab	0.398bc	0.091c	0.195c	0.369c	1.151a
**Genus**												
Collinsella	6.425ab	4.458ab	3.854ab	5.436ab	13.375a	6.446ab	13.023a	1.855b	10.666ab	7.671ab	13.993a	12.911a
Bifidobacterium	2.924cd	2.219d	2.345d	2.552d	8.043c	11.492bc	18.974a	1.738d	7.765c	15.864ab	18.211a	20.182a
Escherichia/Shigella	0.037c	0.062c	0.627c	0.710c	5.804bc	5.010bc	6.604bc	44.176a	7.122bc	7.709b	1.975c	3.876c
Faecalibacterium	12.501a	16.948a	30.136a	21.914a	0.911a	2.419a	2.244a	2.884a	1.936a	2.111a	0.940a	7.602a
Fusobacterium	0.006c	0.016c	0.000c	0.018c	19.176b	6.665c	4.653c	0.665c	18.297b	0.138c	29.702a	5.674c
Romboutsia	1.745b	2.772ab	8.591ab	4.235ab	0.401b	0.159b	11.491a	5.763ab	0.323b	0.199b	6.976ab	10.404a
Dialister	3.981a	3.595a	3.712a	1.920a	0.197a	2.975a	0.817a	5.760a	0.269a	0.415a	0.369a	2.015a
Blautia	15.647a	10.916a	5.698a	3.524a	2.047a	5.963a	2.129a	10.588a	1.757a	3.629a	2.192a	3.243a
Ruminococcus	7.625a	6.869a	2.449a	5.171a	3.055a	0.308a	0.683a	2.375a	2.226a	0.082a	0.547a	0.897a
Agathobacter	3.738a	5.292a	0.233a	7.492a	1.913a	1.768a	0.222a	0.050a	3.981a	1.350a	0.190a	0.060a
Subdoligranulum	3.464a	3.117a	6.954a	4.985a	2.023a	6.850a	10.974a	3.751a	0.803a	3.737a	3.726a	4.683a
Lachnoclostridium	0.057b	0.116b	0.020b	0.194b	8.185a	0.825b	1.260ab	6.819ab	7.779ab	6.610ab	1.295ab	0.923ab
*Erysipelotrichaceae*_UCG-003	1.947ab	2.127ab	0.469b	2.055ab	1.566ab	3.219a	0.638b	0.780ab	3.026ab	3.165a	0.714ab	1.249ab
Bacteroides	0.794b	2.886ab	0.068b	0.650b	5.497a	14.455a	0.208b	0.040b	5.259ab	14.314a	0.190b	0.057b
Dorea	1.707a	1.606a	0.066a	3.909a	1.911a	0.657a	0.136a	0.022a	2.489a	0.308a	0.297a	0.035a
Holdemanella	1.092b	0.887b	0.773b	1.315ab	1.024b	1.296b	2.559a	0.479b	1.346ab	1.508ab	2.165ab	1.996ab
Prevotella	1.533a	2.237a	4.998a	3.764a	0.012a	0.171a	0.174a	0.059a	0.000a	0.129a	0.000a	0.017a
Bilophila	0.013d	0.028d	0.000d	0.034d	2.608ab	1.156abc	0.146cd	0.510bcd	4.640a	2.552ab	0.302cd	0.142d
Acidaminococcus	0.002b	0.000b	0.000b	0.001b	0.896b	4.293ab	0.031b	0.015b	0.718b	6.966a	0.065b	0.031b
Streptococcus	0.813d	0.863d	1.250bcd	1.000cd	1.772bc	0.729d	5.000a	0.784d	1.750bcd	0.777d	3.383b	4.378ab
*Clostridium*_sensu_stricto_1	0.472a	0.887a	2.957a	1.487a	0.102a	0.034a	3.045a	1.766a	0.065a	0.039a	1.769a	3.196a
Fusicatenibacter	0.945a	3.158a	0.050a	1.314a	0.263a	0.359a	0.057a	0.273a	0.564a	0.146a	0.029a	0.008a
Anaerostipes	1.730a	1.647a	0.974a	1.853a	2.310a	0.078a	0.665a	0.653a	3.021a	0.038a	1.315a	0.883a
Intestinibacter	0.327a	0.396a	1.434a	0.875a	0.039a	0.025a	1.829a	0.835a	0.024a	0.027a	1.009a	1.565a
UCG-002	1.793a	2.029a	1.295a	1.490a	0.264a	0.415a	1.122a	0.412a	0.150a	0.537a	0.445a	0.982a
Coprococcus	1.960a	2.759a	0.611a	1.245a	0.280a	0.417a	0.887a	0.470a	0.491a	0.282a	0.643a	1.215a

Table S7- Continued

Monoglobus	1.015ab	0.638b	0.987ab	1.053a	0.161b	0.431b	0.742b	0.101b	0.052b	0.198b	0.274b	0.303b
NK4A214_group	1.213a	1.159a	0.380a	0.595a	0.003a	0.042a	0.022a	0.002a	0.000a	0.028a	0.011a	0.008a
Allisonella	0.009c	0.011c	0.004c	0.007c	4.115a	2.291a	0.142bc	0.254b	2.591a	1.824a	0.192bc	0.052c
Parasutterella	0.026a	0.058a	0.005a	0.051a	0.823a	2.539a	0.052a	0.015a	0.455a	2.699a	0.051a	0.044a
Akkermansia	0.120b	0.137b	0.532ab	0.360b	0.061b	0.059b	0.804a	0.398b	0.043b	0.108b	0.362b	1.149a
Turicibacter	0.146b	0.144b	0.551ab	0.312ab	0.012b	0.011b	0.941a	0.314ab	0.026b	0.018b	0.604ab	0.804a

Lachnospiraceae_NK4A136_group	0.879a	0.595a	0.502ab	0.554a	0.020b	0.000b	0.293ab	0.071b	0.019b	0.000b	0.100b	0.262ab
Sutterella	0.011a	0.035a	0.003a	0.036a	4.338a	3.847a	0.113a	0.034a	2.215a	2.925a	0.018a	0.010a
Parabacteroides	0.180a	0.554a	0.015a	0.086a	1.053a	1.173a	0.050a	0.025a	1.079a	1.046a	0.051a	0.035a
Phascolarctobacterium	0.775ab	0.981ab	1.716ab	0.578ab	0.099b	2.360a	0.112b	0.474b	0.094b	2.300a	0.044b	0.166b
Roseburia	0.533a	0.688a	0.279a	0.756a	0.000a	0.013a	0.106a	0.034a	0.000a	0.000a	0.081a	0.092a
Terrisporobacter	0.082a	0.107a	0.379a	0.220a	0.015a	0.005a	0.474a	0.207a	0.000a	0.000a	0.256a	0.477a
Butyricicoccus	0.886a	0.808ab	0.231ab	0.144b	0.013b	0.019b	0.010b	0.005b	0.390ab	0.003b	0.178ab	0.000b
Alistipes	0.521c	0.857abc	0.071c	0.116c	0.428c	1.881ab	0.017c	0.001c	0.616bc	2.293a	0.126c	0.269c
*Prevotellaceae*_NK3B31_group	0.038b	0.086b	0.012b	0.104b	0.407b	1.910a	0.096b	0.025b	0.023b	0.425b	0.003b	0.012b
Lactobacillus	0.030a	0.017a	0.026a	0.065a	0.051a	0.137a	0.088a	0.012a	0.044a	1.478a	0.104a	0.087a
Desulfovibrio	0.000a	0.000a	0.000a	0.001a	0.159a	0.002a	0.006a	0.000a	0.907a	0.017a	0.043a	0.005a
*Christensenellaceae*_R-7_group	0.621a	0.434a	0.886a	0.686a	0.013a	0.094a	0.107a	0.054a	0.000a	0.085a	0.044a	0.072a
**Species**												
Collinsella aerofaciens	6.424ab	4.453ab	3.854ab	5.436ab	13.372a	6.446ab	13.023a	1.855b	10.665ab	7.666ab	13.993a	12.911a
Fusobacterium ulcerans	0.000b	0.000b	0.000b	0.000b	11.127b	0.000b	4.115b	0.599b	9.795b	0.001b	29.308a	5.435b
Faecalibacterium prausnitzii	5.796a	8.143a	14.710a	10.978a	0.128a	0.291a	0.673a	0.454a	0.194a	0.271a	0.470a	0.783a
Dialister invisus	3.981a	3.595a	3.712a	1.919a	0.197a	2.975a	0.817a	5.760a	0.269a	0.415a	0.369a	2.015a
Ruminococcus bromii	5.410a	4.783a	1.149a	3.350a	2.059a	0.168a	0.325a	0.845a	1.455a	0.048a	0.192a	0.419a
Blautia massiliensis	7.067a	5.110a	0.402a	0.871a	0.192a	2.464a	0.073a	0.046a	0.109a	0.996a	0.028a	0.018a
Bacteroides uniformis	0.342c	0.952bc	0.012c	0.258c	2.756bc	5.739ab	0.090c	0.005c	4.103abc	7.128a	0.104c	0.024c
Dorea longicatena	1.545a	1.415a	0.056a	3.670a	1.801a	0.611a	0.125a	0.022a	2.450a	0.295a	0.297a	0.035a
Bilophila wadsworthia	0.013d	0.028d	0.000d	0.034d	2.608ab	1.156abc	0.146cd	0.510bcd	4.640a	2.552ab	0.302cd	0.142d
Acidaminococcus intestini	0.002b	0.000 b	0.000 b	0.001b	0.661b	3.163ab	0.031b	0.015b	0.495b	5.207a	0.065b	0.031b
Fusicatenibacter saccharivorans	0.917a	3.057 a	0.050 a	1.248a	0.263a	0.359a	0.057a	0.273a	0.564a	0.146a	0.029a	0.008a
Blautia faecis	2.946a	1.647 a	0.049 a	0.288a	0.101a	0.978a	0.036a	0.015a	0.084a	0.439a	0.026a	0.011a
Anaerostipes hadrus	0.989a	0.955 a	0.679 a	1.138a	1.231a	0.058a	0.442a	0.419a	1.594a	0.025a	0.831a	0.613a
Blautia obeum	1.073a	0.713 a	0.597 a	0.297a	0.392a	0.489a	0.408a	1.855a	0.379a	0.480a	0.502a	0.785a
Escherichia coli	0.000b	0.000 b	0.062 b	0.076b	0.663b	0.499b	0.603b	4.672a	0.829b	0.775b	0.170b	0.202b
Intestinibacter bartlettii	0.327a	0.396 a	1.369 a	0.850a	0.039a	0.025a	1.735a	0.815a	0.024a	0.027a	0.971a	1.554a
Coprococcus comes	0.851ab	0.832 ab	0.470 ab	0.377b	0.184b	0.301b	0.802ab	0.418ab	0.357b	0.243b	0.635ab	1.187a
Bacteroides vulgatus	0.064a	0.315 a	0.008 a	0.074a	1.063a	1.929a	0.054a	0.000a	0.641a	1.418a	0.048a	0.025a
Allisonella histaminiformans	0.006b	0.011 b	0.004 b	0.007b	1.753a	0.958a	0.073b	0.138b	1.097a	0.771a	0.132b	0.052b
Bifidobacterium longum	0.228b	0.153 b	0.280 b	0.257b	0.232b	0.233b	1.257a	0.281ab	0.263b	0.214b	0.918ab	0.995ab

Table S7- Continued

Akkermansia muciniphila	0.120b	0.137 b	0.532 ab	0.360b	0.061b	0.059b	0.804a	0.398b	0.043b	0.108b	0.362b	1.149a
Turicibacter sanguinis	0.140b	0.144 ab	0.551 ab	0.305ab	0.012b	0.011b	0.940a	0.297ab	0.026b	0.016b	0.588ab	0.778a
Sutterella wadsworthensis	0.011a	0.024 a	0.003 a	0.028a	4.221a	2.951a	0.113a	0.034a	2.161a	2.061a	0.018a	0.010a
Parabacteroides merdae	0.053a	0.208 a	0.010 a	0.031a	0.786a	0.931a	0.040a	0.005a	0.344a	0.849a	0.010a	0.007a

A two-way ANOVA and Games-Howell post hoc correction were applied. a-c Different letters mean statistically significant differences (*p-value* < 0.05) between samples and times. Data are expressed as the mean relative abundance of taxa. Only taxa with a mean relative abundance > 0.5% are shown, and species properly assigned.
